# Supplementary material for: Medication errors during simulated paediatric resuscitations: a prospective, observational human reliability analysis
Source: BMJ Open. 2019 Nov 25;9(11):e032686. doi: 10.1136/bmjopen-2019-032686 (PMC6886970; doi:10.1136/bmjopen-2019-032686)
Supplement: Supplementary data [file bmjopen-2019-032686supp001.pdf]

Appendices

Appendix 1. Case vignettes, expected medications and acceptable dose ranges

Case 1: Fitting Child

An 8-month-old, 8kg, previously well infant presents in prolonged status epilepticus with a two day history of coryza and one day history of lethargy and fever. On assessment the infant is having a generalised tonic clonic seizure and is febrile.

Teams were expected to treat the child for status epilepticus as per the standard paediatric guidelines, as well as add appropriate antimicrobial cover for presumed meningo-encephalitis. Additionally, all electrolyte abnormalities, including hypoglycaemia (glucose level initially 2.2), should be corrected as they would contribute to seizure activity.

In this case the following drugs were expected to be administered intravenously (in order of clinical priority).

- Lorazepam two doses (0.1mg/kg)
- 10% dextrose for hypoglycaemia (2-5ml/kg) [higher dose accepted as per British National Formulary]
- Phenytoin, loading dose (20mg/kg)
- Rapid Sequence Induction
  - Thiopentone (2-4mg/kg)
  - Suxamethonium (2mg/kg)
- Ceftriaxone (80-100mg/kg)
- Aciclovir (500mg/m^2 = 26mg/kg in this case)
- Clarithromycin (7.5mg/kg)
- Sedation and paralysis post intubation
  - Morphine infusion (10-40mcg/kg/hour)
  - Midazolam infusion (1-6mcg/kg/min)
  - Vecuronium infusion (0.8-3mcg/kg/min)

Case: Sepsis with shock

A 10-month-old, 9kg, previously well and fully immunised infant presents with shock and presumed meningococcal septicaemia. The infant has a two-day history of cough, coryza and fever, and has developed a spreading rash all over his body. On assessment the infant is floppy, only responsive to pain, with a spreading non-blanching purpuric rash all over his torso. He is febrile (40°C) with cold extremities and extremely poor perfusion. He has a sinus tachycardia with an unrecordable non-invasive blood pressure.

Teams were expected to initiate aggressive management of shock with fluids and vasoactive drugs in order to stabilise the infant. Early administration of appropriate antimicrobials was expected, as was the correction of metabolic derangements are also required.

In this case this would mean the following drugs should be administered (in order of clinical priority). Intraosseous access was expected to be established prior to successful intravenous cannulation.

- Ceftriaxone (80-100mg/kg)
- 10% dextrose for hypoglycaemia (2-5ml/kg) [higher dose accepted as per British National Formulary]
- Fluid resuscitation (fluid boluses were not measured for this study)
- Adrenaline infusion (0.1-0.5mcg/kg/min)
    - or
- Dopamine infusion (5-20mcg/kg/min) + adrenaline infusion
- Calcium for hypocalcaemia (0.11mmol/kg)
- Sodium Bicarbonate for acute mixed metabolic/respiratory acidosis (1ml/kg of 8.4%)
- Rapid Sequence Intubation
    - Ketamine (1-2mg/kg)
    - Suxamethonium (2mg/kg)
- Sedation and paralysis post intubation
    - Morphine infusion (10-40mcg/kg/hour)

- Midazolam infusion (1-6mcg/kg/min)
- Vecuronium infusion (0.8-6mcg/kg/min)

Appendix 2. Detailed operational definitions and explanation

Main outcome measures and definitions of error

Medication errors

- **Administrations were classed as <u>dosing errors</u> where:**
  - For drugs given by **injection** (bolus), there was a 10% or greater deviation from the recommended dosing range (DRDR). The DRDR was calculated as the absolute value of the percentage deviation from the recommended dose or dose range.
  - For medications administered as **infusions**, there was a 10% or greater deviation in either the DRDR or the deviation from the recommended dosing rate (DRDRate). The DRDRate was calculated as the absolute value of the percentage deviation from the recommended rate of administration for each medication.
  - For medications administered as **continuous infusions**, there was a 10% or greater deviation from the DRDRate.

- **<u>Timing errors</u>**
  - There is no study to describing acceptable times to medication administration for individual drugs in paediatric emergencies that it is possible to use as a performance benchmark. This lack of clarity is further complicated by the fact that after the successful preparation of a medication to be administered, it is regularly the case that practical issues such as line availability introduce delays. To make an assessment of prolonged administrations, therefore, times to drug 'ready for delivery' (tDRD) were used as a point of reference. The tDRD was calculated as the sum of the elapsed times during which the doctors were seeking medication information in resources with the total nurse-led preparation time. The timings used in this study are shown in more detail in the accompanying figure below.

- tDRD's were considered prolonged where a particular team took greater than 200% of the median tDRD for that specific drug across the entire study sample.
- Drugs given at the wrong time were classed as *'Wrong time'* errors
- Overall delays in tDRD due to clinical reasons as determined by observation, for example interruptions in order to perform critical tasks or give more important medications, were excluded as errors.

Figure 1. Timing definitions used for overall medication errors and individual process steps

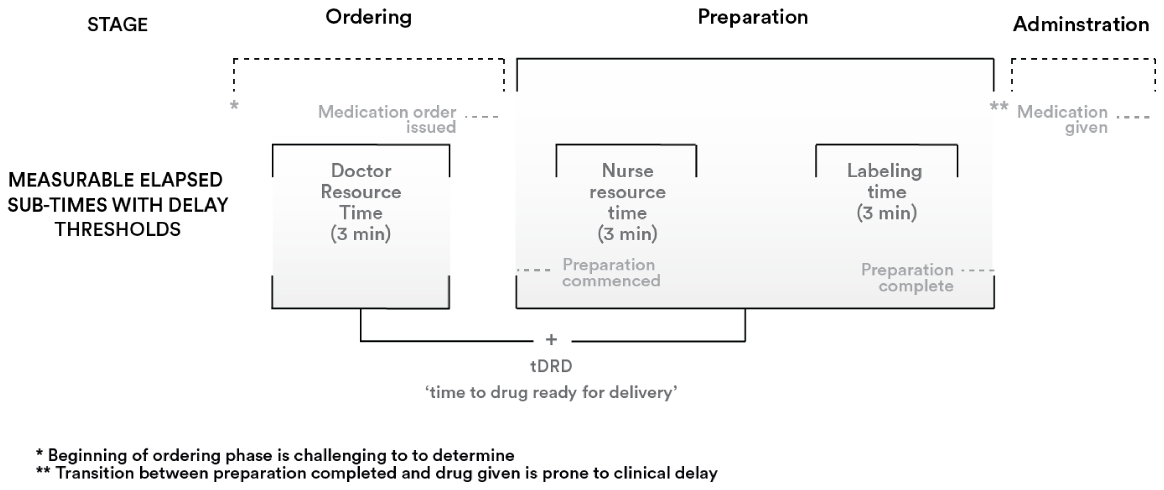

- **Other errors**
  - In addition, medication administrations were considered erroneous overall where
  - There was deviation from the recommended concentration (DRC) of greater than 10% from the suggested concentration in the IV medicines guidance.
  - There was a wrong drug error, a deviation from the suggested route or method of administration.

- All administrations **considered erroneous overall** were presented for severity assessment.

At the level of the task: the HRA / SHERPA framework

1. **Task analysis**

A hierarchical task analysis (HTA) of the procedure for the administration of bolus as well as medications administered by infusion was undertaken, adapting the Lane framework [3] for local emergency practice. The task analysis divided the procedure into three 'doctor-led' and five 'nurse-led' parent tasks, and each of these was broken down hierarchically into exhaustive sub-tasks. The HTA was assessed for face validity by five senior nurses in the study hospital.

2. **Error mode categorisation and assessment**

To understand the steps that were prone to introducing error, we used a generic human error taxonomy based on the SHERPA External Error Modes [4], with the addition of one additional error mode. Each step discrepancy was coded against this framework.

- **Coding thresholds for specific steps**
  - Step discrepancies that **made a contribution of more than 5% DRDR or DRDRate to an overall administration were coded as erroneous**. The lower threshold was chosen to identify causes for errors that were the result of cumulative step discrepancies.
  - There is no guidance with respect to acceptable times for retrieval of medication information from print or electronic resources. Guided by the expert panel, it was decided to consider times for either doctors or the nursing team to retrieve the appropriate information prolonged where they took more than 3 mins at any individual resource.
  - The following were considered the minimum requirements for safe labelling practice:
    - For IV injections: drug name and dose

- For IV infusions and continuous infusions: drug name, dose and strength of prepared mixture after dilution.

Where there were more than two discrepancies at a single step for a specific administration, the nurse evaluator made a subjective assessment of which error had the greater overall consequence and assigned an error mode to that discrepancy only.

3. Downstream or dependent errors

Root cause analysis (RCA) is a technique used to attempt to establish how and why an incident occurred [5]. While this study was not a typical RCA, to capture only 'root-cause' system vulnerabilities, steps where an action was performed correctly, but which perpetuated a previous error were not classed as erroneous. For example, a drug may be prescribed incorrectly, but then drawn up correctly with respect to the prescription. In this case, a prescription error would be recorded, while the drawing up step would not be classed as erroneous. Other errors which are independent of the prescription error, for example failure to double check the medication, were coded as discrepancies if present.

4. Significance assessment for individual step discrepancies

Medication preparation and administration is regularly subject to many minor sub-step discrepancies which may not individually, or even in combination, result in clinically significant error reaching the patient. In order to understand those sub-task errors which contributed to errors overall, significance scores were attributed to each coded sub-step error as follows:

- **No contribution**: discrepancy made no contribution to overall error
- **Minor contribution**: some contribution to overall error. A 5% threshold (for DRDR, DRDRate, DRC) was used to detect task discrepancies contributing in combination to significant errors.
- **Major contribution**: a principally contributory sub-task discrepancy that led directly to the medication error

References

1. Garfield, S., et al., *Measuring the severity of prescribing errors: a systematic review.* Drug Saf, 2013. **36**(12): p. 1151-7.
2. Dean, B.S. and N.D. Barber, *A validated, reliable method of scoring the severity of medication errors.* Am J Health Syst Pharm, 1999. **56**(1): p. 57-62.
3. Lane, R., N.A. Stanton, and D. Harrison, *Applying hierarchical task analysis to medication administration errors.* Applied ergonomics, 2006. **37**(5): p. 669-79.
4. Embrey, D. *SHERPA: A systematic human error reduction and prediction approach*. in *International Topical Meeting Advances in Human Factors in Nuclear Power Systems*. 1986. American Nuclear Society, La Grange Park, Knoxville Tennessee, IL.
5. United States Department of Veteran Affairs., *Root cause analysis*. VA National Center for Patient Safey, 2015. http://www.patientsafety.va.gov/professionals/onthejob/rca.asp
