# Supplementary material for: Medication errors during simulated paediatric resuscitations: a prospective, observational human reliability analysis
Source: BMJ Open. 2019 Nov 25;9(11):e032686. doi: 10.1136/bmjopen-2019-032686 (PMC6886970; doi:10.1136/bmjopen-2019-032686)
Supplement: Author's manuscript [file bmjopen-2019-032686.draft_revisions.pdf]

BMJ Open is committed to open peer review. As part of this commitment we make the peer review history of every article we publish publicly available.

When an article is published we post the peer reviewers' comments and the authors' responses online. We also post the versions of the paper that were used during peer review. These are the versions that the peer review comments apply to.

The versions of the paper that follow are the versions that were submitted during the peer review process. They are not the versions of record or the final published versions. They should not be cited or distributed as the published version of this manuscript.

BMJ Open is an open access journal and the full, final, typeset and author-corrected version of record of the manuscript is available on our site with no access controls, subscription charges or pay-per-view fees (http://bmjopen.bmj.com).

If you have any questions on BMJ Open's open peer review process please email info.bmjopen@bmj.com

BMJ Open

Medication errors during simulated paediatric resuscitations: a prospective, observational human reliability analysis

Journal:	*BMJ Open*
Manuscript ID	bmjopen-2019-032686
Article Type:	Research
Date Submitted by the Author:	08-Jul-2019
Complete List of Authors:	Appelbaum, Nicholas; Imperial College London, Department of Surgery and Cancer, Division of Surgery; Imperial College London, NIHR-Imperial Patient Safety Translational Research Centre<br>Clarke, Jonathan; Imperial College London, NIHR-Imperial Patient Safety Translational Research Centre; Imperial College London, Centre for Mathematics of Precision Healthcare<br>Feather, Calandra; Imperial College London, Department of Surgery and Cancer, Division of Surgery; Imperial College London, NIHR-Imperial Patient Safety Translational Research Centre<br>Franklin, Bryony; Imperial College London, NIHR-Imperial Patient Safety Translational Research Centre; Imperial College Healthcare NHS Trust, Centre for Medication Safety and Service Quality (CMSSQ)<br>Sinha, Ruchi; Imperial College Healthcare NHS Trust, Department of Paediatric Intensive Care, Division of Women and Children's Services<br>Pratt, Phillip; Imperial College London, Department of Surgery and Cancer, Division of Surgery<br>Maconochie, Ian; Imperial College London, Department of Emergency Medicine, Division of Medicine<br>Darzi, Ara; Imperial College London, Department of Surgery and Cancer, Division of Surgery; Imperial College London, NIHR-Imperial Patient Safety Translational Research Centre
Keywords:	PAEDIATRICS, Paediatric A&E and ambulatory care < PAEDIATRICS, Paediatric anaesthesia < PAEDIATRICS

SCHOLARONE™
Manuscripts

Medication errors during simulated paediatric resuscitations: a prospective, observational human reliability analysis

Nicholas Appelbaum MRCS (1, 2, 3), Jonathan Clarke MRCS MPH (2, 3, 8), Calandra Feather RNC BSc (1, 2, 3), Bryony Dean Franklin Pharm PhD (3, 4, 5), Ruchi Sinha MRCPCH (6), Philip Pratt BSc PhD (1, 2), Ian Maconochie FCEM PhD (7) and Ara Darzi FRCS FACS (1, 2, 3)

Author Affiliations:
1 – Department of Surgery and Cancer, Division of Surgery, Imperial College London, UK

2 – Helix Centre for Design in Healthcare, Imperial College London, UK

3 - NIHR-Imperial Patient Safety Translational Research Centre, Imperial College London, UK

4 – UCL Interaction Centre, University College London, UK

5 – Centre for Medication Safety and Service Quality (CMSSQ), Imperial College Healthcare NHS Trust, London, UK

6 – Department of Paediatric Intensive Care, Division of Women and Children's Services, Imperial College London NHS Trust, UK

7 – Department of Emergency Medicine, Division of Medicine, Imperial College London, UK

8 - Centre for Mathematics of Precision Healthcare, Imperial College London

Keywords:
Paediatrics; Paediatric A&E and ambulatory care; Paediatric anaesthesia

Word counts:
Abstract: 252 words
Manuscript: 3938 words

Author contributions:
NA, CF, JC and RS designed the study. RS facilitated the simulations, with NA and CF observing and coordinating. CF analysed the video footage. NA and JC were responsible for the data analysis which was reviewed by PP. BDF and IM advised on error definitions and analysis. NA drafted the study report, which was reviewed by all authors. All authors read and approved the final draft.

Full contact information for corresponding author:

For peer review only

Calandra Feather
Department of Surgery and Cancer
Division of Surgery
Imperial College London
10th Floor, QEQM Building
St Mary's Hospital
Praed Street, London, W2 1NY

Mob: +44 7977185577

Email: c.feather@imperial.ac.uk

Web: www.imperial.ac.uk/ighi
www.helixcentre.com

For peer review only

Medication errors during simulated paediatric resuscitations: a prospective, observational human reliability analysis

Abstract

Introduction: Medication errors during paediatric resuscitation are thought to be common. However, there is little evidence about the individual process steps that contribute to such medication errors in this context.

Objectives: To describe the incidence, nature and severity of medication errors in simulated paediatric resuscitations, and to employ human reliability analysis to understand the contributory role of individual process step discrepancies to these errors.

Methods: We conducted a prospective observational study of simulated resuscitations subject to video micro-analysis, identification of medication errors, severity assessment and human reliability analysis in a large English teaching hospital. Fifteen resuscitation teams of two doctors and two nurses each conducted one of two simulated paediatric resuscitation scenarios.

Results: At least one medication error was observed in every simulated case, and a large magnitude or clinically significant error in 11 of 15 cases. Medication errors were observed in 29% of 180 simulated medication administrations, 40% of which considered to be moderate or severe. These errors were the result of 884 observed discrepancies at a number of steps in the drug ordering, preparation and administration stages of medication use, 8% of which made a major contribution to a resultant medication error. Most errors were introduced by discrepancies during drug preparation and administration.

Conclusions: Medication errors were common with a considerable proportion likely to result in patient harm. There is an urgent need to optimise existing systems and to commission research into new approaches to increase the reliability of human interactions during administration of medication in the paediatric emergency setting.

For peer review only

Strengths and limitations of this study

- This study is one of the first to use HRA methods to link task discrepancies with resultant medication errors, as well as to link these discrepancies directly to potential harm. This effort has demonstrated that a significant fraction of the burden of error in the paediatric emergency drug administration process originates during the preparation and administration phase and that most of these errors are likely to be undetected in clinical practice.
- This study was subject to a number of limitations. Although we went to considerable lengths to replicate the paediatric emergency environment, the simulation environment cannot truly reflect the clinical environment during a genuine emergency.
- Furthermore, this study was conducted at a single site and participants were not blinded to the purpose of the study, so it is potentially subject to preparation bias.
- Participants were recruited from the paediatric emergency unit, intensive care unit and general paediatrics ward and had variable experience of emergency cases. However, all participants worked in clinical units that manage critically ill children.

For peer review only

Introduction

Background

Medication errors are among the leading cause of avoidable harm in healthcare worldwide [1] and up to three times more common in children than in adults.[2] The paediatric emergency environment, characterised by urgency and fraught with interruptions, is one of the clinical areas most vulnerable to error. Medication administration in emergencies is complex as it requires successful interactions between different teams of medical and nursing staff, as well as between individual members of these teams. An additional challenge relates to obtaining relevant medication information and translating this into the required dose and concentration of the correct drug to be administered by the correct route for the clinical indication, all

For peer review only - http://bmjopen.bmj.com/site/about/guidelines.xhtml

in a necessarily short space of time. Medication errors in general, and medication administration errors in particular, are both under-detected and under-reported, such that little is known of their incidence or impact during resuscitation. However, medication errors have been reported in 7 out of 10 simulated paediatric resuscitations,[3] with other recent simulation studies suggesting 26% [4] to 70% [5] of administered medicines being given at the wrong dose. Laboratory studies analysing syringes prepared for anaesthetic use have found at least 15% to be greater than 20% discrepant from the intended drug concentration.[6]

The broader, systems view of medical error, heralded by the Institute of Medicine's "To Err is Human" report, saw the widespread adoption of Reason's organisational accident model [7] in healthcare. More recently, human reliability analysis (HRA) techniques, previously commonplace only in other high risk industries, have become increasingly used in healthcare.[8,9] HRA is based on the understanding that neither humans nor systems can be error-proof, and asserts that to improve safety and reliability, a thorough analysis of system vulnerabilities at a task level is needed, taking into account human-human and human-machine interactions.[10,11] Medication safety researchers have previously used a HRA technique, the systematic human error reduction and prediction approach (SHERPA), to identify system vulnerabilities in ward-based medication administration,[12] anaesthesia[13] and general surgery.[14] However, this approach has not been used quantitatively in medication safety research and has not been applied to paediatric resuscitation.

Our objectives were to describe the incidence, nature and severity of medication errors in simulated paediatric resuscitations, and then use HRA to understand the contributory role of individual process step discrepancies with a focus on those contributing to large magnitude and/or clinically significant errors.

Methods

Study design and setting

This prospective observational study was conducted April to November 2017 in a medical simulation facility within a large teaching hospital. The hospital has a paediatric emergency department (seeing 27,000 children each year) and a

For peer review only

comprehensive paediatric inpatient service (admitting 5,000 each year). The hospital used electronic prescribing in the inpatient setting, but during resuscitations, medications were more commonly ordered on paper prescription charts. We recruited resuscitation teams of four clinicians, that were randomised to participate in one of two standardised simulated paediatric resuscitation scenarios. The study was approved by the Health Research Authority and the hospital concerned. NHS ethics approval was not required. Participants gave written informed consent.

Patient and Public Involvement

The research team held a workshop with parents to get their feedback on the proposal, develop the PPIE plans, and identify future important areas for research. We actively sought attendees through INVOLVE's "People in Research" website, social media and Imperial College London's existing networks. Our team has also participated in a PPIE event run by the Royal College of Paediatrics and Child Health, in collaboration with MedsIQ (http://www.medsiq.org) and Medicines for Children (http://www.medicinesforchildren.org.uk), two UK-based paediatric medication safety initiatives.

Participants

Eligible participants were a convenience sample of medical and nursing staff from the departments of paediatrics and paediatric emergency medicine at the study hospital. Participants were assigned into teams comprising a senior doctor (a specialist registrar, with at least a year of prior experience as a registrar), a junior doctor, a senior nurse (with at least 5 years' nursing experience) and a junior nurse.

Clinical scenarios

The two scenarios were:

A. Prolonged status epilepticus in an 8-month-old, 8kg child
B. Presumed meningococcal sepsis in a 10-month-old, 9kg child

The two scenarios (**Appendix 1)** were designed by a collaboration of paediatric nurses, emergency physicians, intensivists, general paediatricians, and anaesthetists. Face validity was established by an independent expert panel of six, with

For peer review only - http://bmjopen.bmj.com/site/about/guidelines.xhtml

representation from each of these professional groups, including two lead paediatric clinical nurse educators. The two scenarios were determined to be similarly demanding.

A simulated paediatric resuscitation bay was created. The mannequin used was a SimBaby Version 2 (Laerdal Medical, Stavanger, Norway), and the syringe pump stack consisted of Alaris PK MK4 units (Becton Dickinson, Franklin Lakes, USA). All relevant print materials (e.g. formularies and protocols) and hospital information technology systems were available. Participants were requested to prescribe, prepare and administer medications exactly as per usual practice, to use mobile applications or websites as they would in clinical practice, and to telephone specialist colleagues if required. A paediatric intensivist, the hospital lead for paediatric simulation, ran the scenarios. She provided standardised clinical information as live feedback and answered questions regarding the child's response to treatment or their current condition when needed.

Data sources and measurement

A Scotia Medical Observation System (Smots, Scotia UK, Edinburgh, UK), with two 3-axis, ceiling-mounted video cameras, and three mobile, high-definition cameras equipped with boom microphones, was used. Both nurses in each team wore head-mounted high-definition video cameras (GoPro Inc, California, USA). The video recordings were analysed by a research nurse with ten years' experience in paediatric intensive care.

Outcome measures

We used the term 'medication error' to describe an overall error with respect to a particular drug administration as a whole, and the term 'discrepancy' to refer to observed deviations at the level of the individual task. A discrepancy may or may not lead to a medication error.

Variation in clinician practice and human adaption to the complex process of medication ordering, preparation and administration typically results in many minor task discrepancies that may not individually, or even in combination, result in a medication error or patient harm.[15] To identify the most important task discrepancies,

For peer review only

we assessed all observed discrepancies to establish the extent to which they may or may not have contributed to any resultant medication errors.

Figure 1 summarises the study objectives and associated analyses.

Medication errors

Medication errors included any errors in dose, administration rate, concentration, drug, route or method of administration, timing, or delay in administration. Operational definitions for each of these are given in **Appendix 2.** Briefly, dosing errors were defined as a greater than 10% deviation from the recommended dosing range (DRDR) [16] at the study site. Any deviation from the recommended rate of administration (DRDRate) was calculated in a similar manner and deviations of more than 10% were considered to be medication errors. Deviations from the recommended concentration (DRC) of greater than 10% from the concentration specified in local guidance were also included as medication errors.

To identify delayed administrations, the time taken for the dose to be 'ready for delivery' (tDRD) was calculated as the time for the doctors to obtain any medication information required plus the nurse-led preparation time. The time to be ready for deliver was considered 'prolonged' when a particular team took more than double the median time for that specific drug across the entire study without clinical cause for the delay as determined by the nurse assessor. For example, if a medication administration was interrupted to reassess the patient clinically or to administer another medication as a priority, a prolonged tDRD would be excluded as an error on clinical grounds.

Severity assessment

There are few validated tools that can be used to assess the severity of medication errors without knowledge of patient outcomes and that are thus usable in simulated studies [17]. One of these tools is that of Dean and Barber, based on 4-5 experts independently assessing each error on a 0 to 10 scale, and their mean score used as an index of severity. Mean scores under 3 suggest errors of minor severity, those between 3 and 7 as moderate and those greater than 7 as severe.[18] We therefore

For peer review only - http://bmjopen.bmj.com/site/about/guidelines.xhtml

used this approach, with two paediatric intensivists, one paediatric anaesthetist, one senior critical care nurse, and one senior clinical pharmacist assessing each error.

Discrepancies at the level of the task

A hierarchical task analysis (HTA) was developed based on a similar framework for ward-based medication administration[12] and assessed for face validity by five senior nurses in the study hospital. A generic human error taxonomy, based on SHERPA external error modes[19] with one additional error mode, 'information not sought', was used to code observed discrepancies against the HTA.

Where there were more than two discrepancies at a single step for a specific administration, the nurse assessor made a subjective assessment of which had the greater overall consequence, and assigned an error mode (Figure 2) to that discrepancy only. To capture 'root-cause' system vulnerabilities, steps where an action was performed correctly, but which perpetuated a previous medication error, were not classed as discrepancies.

Significance assessment of task discrepancies

All task discrepancies were classified by the nurse assessor according to the contribution made by the discrepancy as follows:

No contribution: the discrepancy did not contribute to a medication error

Minor contribution: some contribution made to a medication error

Major contribution: the task discrepancy led directly to a medication error

Data management and analysis

Medication errors were described according to the type of error, method of administration and stage of the medication use process in which the error occurred. Error rates were calculated using the total number of applicable administrations as the denominator.

For peer review only - http://bmjopen.bmj.com/site/about/guidelines.xhtml

Step discrepancies were presented as counts grouped by task and contribution to medication error. Discrepancy rates were calculated as the percentage of discrepancies that made a major, minor or no contribution to an error, with the number of observed discrepancies at each process step as the denominator. Of those discrepancies making major contributions to medication errors, the proportion that led to clinically significant errors (severity score > 3) and/or large magnitude errors (DRDR or DRDRate > 25%) was also calculated.

There is no literature that quantifies the extent to which a step discrepancy having a minor or major contribution to an error is of greater significance than a step discrepancy that makes no contribution to an error. For a weighted, 'heat map' HRA analysis, it was therefore necessary to attribute different weights to discrepancies that resulted in error to those that did not. Sub-step discrepancies were therefore weighted, agreed by expert panel, as follows:

- No contribution: weight = 1
- Minor contribution: weight = 10
- Major contribution: weight = 40

The total weighted significance score for each step was thereby calculated for each error mode.

Inter-observer reliability

One of the 15 simulations was re-analysed by an additional independent nurse. Spearman's rank correlation coefficient was calculated for continuous variables and Cohen's Kappa for categorical variables.

Results

Data were collected during 15 simulations according to participant availability, eight for prolonged seizures, and seven for meningococcal sepsis. Participants comprised 30 doctors and 30 nurses (**Table 1)**, each of whom completed one simulation. Inter-observer reliability analysis revealed perfect agreement for categorical variables, and

For peer review only - http://bmjopen.bmj.com/site/about/guidelines.xhtml

high agreement for continuous variables with a Spearman's rank correlation coefficient 0.957 or above.

Table 1. Characteristics of study population

	Overall	Doctors	Nurses
**Total number of participants**	60	30	30
**Age †**			
Median (range)	30 (23-51)	30.5 (23-44)	28 (23-51)
**Gender (%)**			
Female	52 (87%)	23 (77%)	29 (97%)
Male	8 (13%)	7 (23%)	1 (3%)
**Years in clinical practice (n, %)**			
0-5	32 (53%)	11 (37%)	21 (70%)
6-10	20 (33%)	16 (53%)	4 (13%)
11-15	5 (8%)	3 (10%)	2 (7%)
16-20	1 (2%)	0 (0%)	1 (3%)
>20	2 (3%)	0 (0%)	2 (7%)
**Years in paediatric practice (n, %)**			
0-5	37 (62%)	16 (53%)	21 (70%)
6-10	16 (27%)	12 (40%)	4 (13%)
11-15	5 (8%)	2 (7%)	3 (10%)
16-20	1 (2%)	0 (0%)	1 (3%)
>20	1 (2%)	0 (0%)	1 (3%)

† Age data was omitted for five participants

For peer review only

Medication errors

Participants conducted 180 medication administrations. Overall, errors were observed reaching the patient for 52 drug administrations (29%) and at least once in every simulation. Of these errors, 30 (58%) were assessed as being of minor severity, 16 (31%) as moderate and 6 (12%) as severe. There were 27 large magnitude errors (52% of all errors), in which the DRDR / DRDRate was greater than 25%. Of all erroneous administrations that reached the patient, only two (4%) were noticed by staff after administration and therefore may have been reported in clinical practice. A detailed error analysis is provided in **Table 2** and description of the ten most severe errors in **Table 3**.

Hierarchical task analysis

The full HTA is shown as **Figure 3** and shows all steps assessed in the paediatric emergency drug administration process.

Errors and discrepancies by stage of medication use and process sub-step

Overall, 884 step discrepancies were observed, excluding dependent downstream discrepancies after an initial discrepancy. Of these 884 step discrepancies, 174 (20%) were linked to a medication error, with 70 (8%) assessed as making a major contribution to an error, 104 (12%) making a minor contribution, and 710 (80%) making no contribution.

Figure 2 shows the significance-weighted HRA data represented as a heat-map demonstrating the relative contributions of discrepancies at each step and by each error mode to medication errors. **Table 4** summarises the discrepancy counts per step as well as the percentage of both large magnitude and clinically significant errors with a major contribution made at each HTA step.

Errors and discrepancies during medication ordering

We observed 170 discrepancies during the ordering phase. Five of the 22 clinically significant medication errors were due to discrepancies during medication ordering, with three of these due to incorrect dose information retrieval from the British National Formulary for Children. The majority of the remaining discrepancies (136) were due to incomplete verbal medication orders based on which drug preparation commenced, two of which resulted in medication errors. Of the 180 written medication orders examined, there were six discrepancies observed, all of which were corrected by the clinicians and therefore made no contribution to any dosing errors, but did result in one delayed administration.

For peer review only

Table 2. Incidence, nature and severity of errors, presented by phase and type of error and method of administration

	Number of errors	Incidence as % of total administrations (n=180)	Incidence as % of administrations by method of administration	Severity assessment: Minor (severity score < 3) (n)	Severity assessment: Moderate (severity score 3 - 7) (n)	Severity assessment: Severe (severity score > 7) (n)	Severity assessment: Mean severity score
**Any error**	**52**	**29%**		**30**	**16**	**6**	**3.2**
By stage of medication use and error type							
**Prescription errors**	**8**	**4%**		**2**	**4**	**2**	**5.1**
Wrong dose	8	4%	-	2	4	2	5.1
Wrong route	1	1%	-	-	-	1	-
Wrong drug	-	-	-	-	-	-	-
**Preparation and administration errors**	**40**	**22%**		**25**	**12**	**5**	**3.1**
Wrong drug	1	1%	-	-	-	1	-
Wrong dose prepared	10	6%	-	6	3	1	2.8
Wrong diluent / concentration	20	11%	-	12	6	2	3.0
Wrong rate (infusions)	11	6%	-	4	5	2	4.2
Wrong route / method	9	5%	-	5	3	1	3.6
Wrong time	1	1%	-	-	-	1	-
By method of administration and error magnitude							
**Bolus doses (n = 77 in total)**							
**Any error**	**24**	**-**	**31%**	**13**	**7**	**4**	**3.6**
Dose error, DRDR† > 10%	7	-	9%	-	5	2	5.0
Dose error, DRDR† > 25%	6	-	8%	-	4	2	5.7
Delayed administration, dose correct	5	-	7%	2	2	1	4.2
Delayed administration, dose error	-	-	-	-	-	-	-
**Intermittent infusions (n = 48 in total)**							
**Any error**	**18**	**-**	**38%**	**10**	**7**	**1**	**3.2**
Total dose error, DRDR† > 10%	5	-	10%	3	2	-	3.7
Total dose error, DRDR †> 25%	3	-	6%	1	2	-	5.2
Rate error, DRDRate‡ > 10%*	8	-	17%	2	5	1	4.6
Rate error, DRDRate‡ > 25%*	6	-	13%	-	5	1	6.0
Delayed administration, correct dose and rate	2	-	4%	2	-	-	1.4
Delayed administration, incorrect dose or rate	1	-	2%	-	1	-	-
**Continuous infusions (n = 55 in total)**							
**Any error**	**10**	**-**	**18%**	**7**	**2**	**1**	**2.2**
Delivery rate error, DRDRate‡ > 10%	5	-	9%	2	2	1	3.4
Delivery rate error, DRDRate‡ > 50%	5	-	9%	2	2	1	3.4
Delayed administration, correct delivery rate	1	-	2%	1	-	-	-
Delayed administration, with incorrect delivery rate	1	-	2%	-	1	-	-

More than one error type can occur in one medication administration and a single medication error may meet more than one criterion, so individual error types do not sum to the total by stage or method. Other error types (for example diluent errors) are included in the 'any error' counts, but are not presented as sub-counts.

*Rate errors are only shown for intermittent infusions where the delivery rate error is due to a pump-programming error.

† DRDR (deviation from recommended dosing range) = absolute value of the percentage difference from the recommended dose or dose range

‡ DRDRate (deviation from recommended dosing rate) = absolute value of the percentage difference from the recommended rate of administration

For peer review only

Table 3. Details of the ten most severe medication errors by severity score

Rank	Medication	DRDR †	DRDRate ‡	Other error	Severity score	Error detail	Error cause
1	Thiopentone	900%	-	-	8.8	320mg rather than 32mg given as IV bolus	Doctors asked for two doses of thiopentone to be prepared which were ordered at 32mg each. Nurse read phenytoin dose, which was 160mg, written on the line above the thiopentone order on the medication chart. Two boluses of 160mg rather than 32mg were prepared and administered.
2	Calcium chloride	789%	-	-	8.6	8.8mmol rather than 0.99mmol given as IV bolus	Incorrect dose for indication selected and prescribed from British National Formulary (BNF)
3	Thiopentone	-	-	Timing error	8.2	Given prior to anaesthetist ready	Medication prepared and administered prior to medical and anaesthetic team being ready to manage airway and breathing
4	Dextrose	-	-	14 minutes taken to administer	7.5	825% median time for dextrose. Hypoglycaemic patient, glucose = 2.2	Excessive time spent working out required volume to administer due to confusion caused when checking concentration information across multiple resources
5	Adrenaline	-	-88.9%	18 minutes taken to administer	7.5	286% median time for adrenaline infusions	Team unfamiliar with prescribing and preparing adrenaline infusion, time spent accessing multiple resources
6	Phenytoin	-	300%	-	7.2	Correct dose given undiluted at four times the recommended administration rate (4mg/kg/min rather than 1mg/kg/min)	Calculation error when setting up rate on pump, administered over 5 minutes instead of 20 minutes. Undiluted phenytoin infusions are not in accordance with local policy but this was not clearly indicated in the IV administration guidance
7	Phenytoin		-99.2%	-	6.9	Correct dose given too slowly (0.0078mg/kg/min rather than 1mg/kg/min)	Calculation error when setting up rate on pump
8	Aciclovir	-45.5%	163.3%	-	6.8	105mg rather than 210mg given as infusion and run over 20 mins instead of an hour	Incorrect dose chosen for indication from BNF and administered over 20 mins instead of 1 hour. Correct dose was on following page of BNF
9	Aciclovir	-45.5%	-	-	6.2	105mg instead of 210mg	Incorrect dose selected for indication from BNF. Correct dose was on following page of BNF
10	Aciclovir	-	high	-	5.7	Given as a neat bolus rather than being diluted and infused	Administered as bolus instead of as an infusion over 1 hour

† DRDR (deviation from recommended dosing range) = percentage difference from the recommended dose or dose range

‡ DRDRate (deviation from recommended dosing rate) = percentage difference from the recommended rate of administration

For peer review only - http://bmjopen.bmj.com/site/about/guidelines.xhtml

Table 4. Number, frequency and relationship of discrepancies to resultant errors with sub-analysis of major discrepancies that resulted in large magnitude and clinically significant errors

		Relationship to resultant errors				
				Discrepancies which made a major contribution to a medication error		
**Stage of deviation**	**Total discrepancies (n)**	**No contribution n (%*)**	**Minor contribution n (%*)**	**Major contribution, total (n, %*)**	**Discrepancies that resulted in clinically significant errors (n, %*) †**	**Discrepancies that resulted in large magnitude errors n (%*) ‡**
**OVERALL**	**884**	**710 (80.3)**	**104 (11.8)**	**70 (7.9)**	**33 (47.1)**	**31 (44.3)**
**ORDERING PHASE**	**170**	**159 (93.5)**	**2 (1)**	**9 (5)**	**5 (23)**	**6 (22)**
**Determine dose**	**29**	**21 (72)**	**1 (3)**	**7 (24)**	**5 (23)**	**6 (22)**
Dose from memory	4	2 (50)	-	2 (50)	-	1 (4)
Dose from formulary	10	6 (60)	1 (10)	3 (30)	3 (14)	3 (11)
Dose from other resource	11	10 (91)	-	1 (9)	1 (5)	1 (4)
Dose calculation	4	3 (75)	-	1 (25)	1 (5)	1 (4)
**Issue order**	**141**	**138 (97.9)**	**1 (1)**	**2 (1)**	**-**	**-**
Issue verbal order	136	134 (98.5)	1 (1)	1 (1)	-	-
Issue written order	5	4 (80)	-	1 (20)	-	-
**PREPARATION PHASE**	**588**	**456 (77.6)**	**89**	**43 (7)**	**17 (77.3)**	**13 (48)**
**Check order**	**19**	**6 (32)**	**9 (47)**	**4 (21)**	**3 (14)**	**1 (4)**
Check drug name	1	-	-	1 (100)	1 (5)	1 (4)
Check dose for indication	15	5 (33)	8 (53)	2 (13)	1 (5)	-
Check calculation	1	-	1 (100)	-	-	-
Check route and timing	2	1 (50)	-	1 (50)	1 (5)	-
**Preparation, actual**	**310**	**263 (84.8)**	**8 (3)**	**39 (13)**	**14 (64)**	**12 (44.4)**
Find correct medication and strength of vial	2	-	2 (100)	-	-	-
Check intravenous administration guidance	42	22 (52)	1 (2)	19 (45)	9 (41)	6 (22)
Check ampoule	1	-	1 (100)	-	-	-
Check reconstitution fluid and volume	7	3 (43)	1 (14)	3 (43)	-	-
Convert milligrams to millilitres	7	2 (29)	-	5 (71)	3 (14)	4 (15)
Find correct syringe and draw neat volume	8	3 (38)	-	5 (63)	1 (5)	1 (4)
Determine correct concentration for route	2	2 (100)	-	-	-	-
Calculate further diluent volume and dilute	12	4 (33)	1 (8)	7 (58)	1 (5)	1 (4)
Label syringe with drug name, dose and concentration if appropriate	231	227 (98.3)	4 (2)	-	-	-
**Double checking**	**259**	**187 (72.2)**	**72 (27.8)**	**-**	**-**	**-**
Check reconstitution	9	5 (57)	4 (44)	-	-	-
Check dose and neat volume	69	48 (70)	21 (30)	-	-	-
Check diluent volume and total volume separately	86	63 (73)	23 (27)	-	-	-
Check ampoule + expiry	29	26 (90)	3 (10)	-	-	-
Check rate / method	66	45 (68)	21 (32)	-	-	-
**ADMINISTRATION PHASE**	**28**	**8 (29)**	**2 (7)**	**18**	**11 (50)**	**12 (44.4)**
Boluses: inject	2	1 (50)	1 (50)	-	-	-
Infusions: determine run rate	8	-	1 (13)	7 (88)	5 (23)	5 (19)
Determine Y-site compatibility	2	2 (100)	-	-	-	-
Determine delivery rate for continuous infusions	7	3 (43)	-	4 (57)	3 (14)	4 (15)
Program infusion pump	9	2 (22)	-	7 (78)	3 (14)	3 (11)
**RECORDING**	**88**	**88 (100)**	**-**	**-**	**-**	**-**

* The denominator for percentages is the total number of discrepancies in each row

† Number and percentage of clinically significant errors (severity score > 3) with major contributory discrepancies made at each specific step, of a total of 22 clinically significant errors.

‡ Number and percentage of large magnitude errors [deviation from recommended dosing range (DRDR) or deviation from recommended dosing rate (DRDRate) > 25%] with major contributory discrepancies made at each specific step, of a total of 27 large magnitude errors

For peer review only - http://bmjopen.bmj.com/site/about/guidelines.xhtml

Errors and discrepancies during medication preparation

During medication preparation, 310 discrepancies were observed, representing 35% of all observed discrepancies. These contributed to one medication error involving the wrong drug, ten dose errors and 20 diluent or dilution errors. The retrieval of preparation and administration information from the online IV medications guide was the step mostly likely to contribute to medication errors during medication preparation, with 42 discrepancies (19 major contribution to a medication error, one minor), resulting in nine clinically significant medication errors (severity score > 3). The retrieval of incorrect information and taking an excessively long time to identify the correct information within the guidance were the most common discrepancy error modes.

There were seven discrepancies when converting milligrams to millilitres of undiluted drug, and 18 discrepancies (six making a major contribution) when, after having made the correct calculations, nurses withdrew either the incorrect amount of undiluted drug or the incorrect amount of diluent into the syringe.

Double-checking

Overall, 259 discrepancies were observed during the double-checking phase, 72 (28%) of which made a minor contribution to a medication error. Checking the route and method (e.g. infusion or bolus) of administration was the most frequently omitted.

We observed 29 medication errors that were made during medication ordering and preparation but had not been yet administered to the patient at the point of double checking. These errors were thus potentially 'interceptable' but all ultimately reached the patient. Of these errors, in 14 cases, the double-checking interaction between the nurses included the incorrect step, but failed to identify it as incorrect.

Errors and discrepancies during the administration phase

Of all observed discrepancies, only 28 (3%) occurred during administration. These resulted in 11 wrong rate errors (five moderate, two severe), nine wrong method errors

For peer review only - http://bmjopen.bmj.com/site/about/guidelines.xhtml

(three moderate, one severe) and one severe wrong time error. Discrepancies during the administration phase constituted a third of all discrepancies that made a major contribution to a clinically significant error.

Infusions in particular were prone to administration errors. Of the 17 discrepancies observed during infusion rate calculations or when programming the infusion pump for intermittent infusions, 14 were of major consequence, and accounted for 23% of all clinically severe errors. Seven discrepancies (four making a major contribution to a medication error) occurred when determining the delivery rate for continuous infusions.

Discussion

This prospective observational study is the first in paediatric emergency medicine to include a quantitative HRA, allowing identification of the task discrepancies with the greatest contribution to medication error. We identified at least one medication error in all 15 simulations, and a large magnitude or clinically significant error in 12 of these.

Comparison with previous literature

Historical heterogeneity of the definitions of medication error and the variability in reporting metrics make comparison with previous literature difficult.[20,21] Additionally, there are few simulated studies and no relevant clinical studies in paediatric resuscitation, making comparator data scarce.

Prescribing error rates in the emergency setting have been reported to be 10.1% and 16% [22,23] of all orders; our study reports a lower rate of 5%, although this difference may be at least partly due to different error definitions. Our study instead suggests that preparation and administration errors may be more common. Other simulated studies have reported error rates for the administration of intravenous bolus medication of between 15.5% and 26.5% [3,4,24]; in our study, it was 31%. The referenced studies, however, reported only on dose errors, and not any other medication error types. Only seven out of the 24 medication errors we observed for bolus doses were dose errors. Medications given by intermittent infusion were the most error-prone in our study.

For peer review only - http://bmjopen.bmj.com/site/about/guidelines.xhtml

For peer review only

There are no studies that investigate emergency administration of intermittent infusions in sufficient detail to provide a basis against which to compare this finding.

Medications given by continuous infusion are potentially the most complicated in paediatric emergencies. In addition to the preparation steps for intermittent infusions, staff generally have to convert infusion rates in milligrams or micrograms per kilogram per minute to infusion rates of millilitres per hour. A recent trial of a digital application reported errors in 70% of continuous vasopressor infusions [5] in the control arm. However, despite the increased cognitive demand, we observed the lowest incidence of medication error for continuous infusions, at 18%. Administration of continuous infusions in our hospital seemed to be relatively well supported by an online / paper tool [25].

Implications for research and practice

This study highlights the need for research to optimise clinicians' use of electronic resources containing medication preparation and administration information. We were not able to pinpoint the precise steps at which the current electronic intravenous medications guidance system in the study hospital proved vulnerable to misinterpretation. Research to further understand the steps that need attention may serve as a useful basis from which to refine, and if needed, redesign such systems.

This study reaffirms that performing complex arithmetic in high-stress clinical environments is a considerable contributor to medication error.[26] With the purpose of addressing medication safety in paediatric resuscitation, the literature has been dominated by studies looking at 'resuscitation aids', most commonly length-based tapes.[16,27,28] These aids couple weight estimation with a suggested dose for a limited number of medications, but without providing preparation and administration support. It is not likely that length-based tapes would have decreased the rate of medication error in this study. Further clinical research is required to determine the effectiveness of new digital tools that do support preparation and administration, such as those that have shown promising results in simulated studies.[5,29]

Human factors methods have been used in other high-risk industries to define system vulnerabilities for building safer systems. By using quantitative HRA, this study

For peer review only - http://bmjopen.bmj.com/site/about/guidelines.xhtml

provides evidence for the prioritisation of research efforts directed towards new interventions to address the most important system weaknesses.

In terms of implications for practice, one of the most unexpected findings in this investigation was the uncovering of 'purely mechanical' task discrepancies resulting in medication errors. During drug preparation, clinicians were observed drawing up incorrect volumes of medications or diluents even though all calculations were correct. This suggests that efforts seeking to address medication safety in cognitively demanding environments using clinical education strategies or contemporary technologies must do so without disregarding the seemingly 'simplest' aspects of drug preparation. The reliability of information exchanges between healthcare professionals similarly needs improvement. Verbal medication orders in particular are inconsistent and error-ridden. Particular attention should be paid to medication orders given verbally in the emergency setting, using approaches such as the recipient verbally confirming the medication and dose being prepared. More importantly however, there is an urgent need for research to explore how to bring greater effectiveness to checking and double-checking more broadly. These are steps intended to defend patients from error, but which are too often ineffective.

Conclusions

Overall, we identified errors in 29% of all simulated medication administrations, only two of which were detected by participants, with 40% of these likely to result in moderate or severe harm. HRA revealed a number of error-prone steps, many of which occurred during preparation and administration of correctly ordered medications. The task most likely to result in erroneous medication administration was ineffective retrieval of correct medication preparation and administration instructions from intravenous medication guidance.

This study has highlighted an urgent need to optimise existing systems and to commission new approaches to increase the reliability of human interactions with the emergency medication administration process.

For peer review only - http://bmjopen.bmj.com/site/about/guidelines.xhtml

Acknowledgements

We would like to thank all clinical staff who participated in the simulations. It is our hope that the enthusiasm and extraordinary open-mindedness our dedicated and deeply competent colleagues demonstrated in making this study possible will lead to safer systems and innovations in paediatric medication safety.

This study was funded by the NIHR-Imperial Patient Safety Translational Research Centre, with infrastructure support from the NIHR Imperial Biomedical Research Centre. Support was also received from the Resuscitation Council (UK).

Ethical approval: The Health Research Authority approved this study.

Conflicts of interest

The Helix Centre at Imperial College London is leading an effort in collaboration with the British National Formulary developing digital tools in an attempt to improve paediatric medication safety. NA has written two patents describing syringe labelling techniques in medication safety. BDF supervises a PhD student part funded by a supplier of hospital electronic health record systems, and has received funding from Pfizer for delivering teaching at a one-off symposium on medication safety.

We confirm that we have given due consideration to the protection of intellectual property associated with this work and that there are no impediments to publication, including the timing of publication, with respect to intellectual property. In so doing we confirm that we have followed the regulations of our institution concerning intellectual property.

For peer review only

References

1. World Health Organization. Medication without harm. *B World Health Organ.* 2017;95(5):317-317.
2. Wong IC, Ghaleb MA, Franklin BD, Barber N. Incidence and nature of dosing errors in paediatric medications: a systematic review. *Drug Saf.* 2004;27(9):661-670.
3. Kozer E, Seto W, Verjee Z, et al. Prospective observational study on the incidence of medication errors during simulated resuscitation in a paediatric emergency department. *Bmj.* 2004;329(7478):1321.
4. Moreira ME, Hernandez C, Stevens AD, et al. Color-Coded Prefilled Medication Syringes Decrease Time to Delivery and Dosing Error in Simulated Emergency Department Pediatric Resuscitations. *Ann Emerg Med.* 2015;66(2):97-106.e103.
5. Siebert JN, Ehrler F, Combescure C, et al. A Mobile Device App to Reduce Time to Drug Delivery and Medication Errors During Simulated Pediatric Cardiopulmonary Resuscitation: A Randomized Controlled Trial. *J Med Internet Res.* 2017;19(2).
6. Stucki C, Sautter AM, Wolff A, Fleury-Souverain S, Bonnabry P. Accuracy of preparation of i.v. medication syringes for anesthesiology. *Am J Health-Syst Pharm.* 2013;70(2):137-142.
7. Reason J. *Human Error.* Cambridge: University of Cambridge; 1990.
8. Embrey D. Application of SHERPA to Predict and Prevent Use Error in Medical Devices. Paper presented at: International Symposium on Human Factors and Ergonomics in Healthcare: Advancing the cause2014.
9. Lyons M AS, Woloshynowych M, Vincent C. Human reliability analysis in healthcare: A review of techniques. *International Journal of Risk & Safety in Medicine.* 2004;16:223-237.
10. Kirwan B AL. *A guide to task analysis.* London: Taylor and Francis; 1992.
11. Authority NOPSaEM. HUMAN RELIABILITY ANALYSIS. 2018; https://www.nopsema.gov.au/resources/human-factors/human-reliability-analysis/.
12. Lane R, Stanton NA, Harrison D. Applying hierarchical task analysis to medication administration errors. *Applied ergonomics.* 2006;37(5):669-679.
13. Phipps D, Meakin GH, Beatty PC, Nsoedo C, Parker D. Human factors in anaesthetic practice: insights from a task analysis. *Br J Anaesth.* 2008;100(3):333-343.
14. Joice P, Hanna GB, Cuschieri A. Errors enacted during endoscopic surgery--a human reliability analysis. *Appl Ergon.* 1998;29(6):409-414.
15. Lyons I, Furniss D, Blandford A, et al. Errors and discrepancies in the administration of intravenous infusions: a mixed methods multihospital observational study. *BMJ Qual Saf.* 2018.
16. Shah AN, Frush K, Luo X, Wears RL. Effect of an intervention standardization system on pediatric dosing and equipment size determination: a crossover trial involving simulated resuscitation events. *Arch Pediatr Adolesc Med.* 2003;157(3):229-236.
17. Garfield S, Reynolds M, Dermont L, Franklin BD. Measuring the severity of prescribing errors: a systematic review. *Drug Saf.* 2013;36(12):1151-1157.
18. Dean BS, Barber ND. A validated, reliable method of scoring the severity of medication errors. *Am J Health Syst Pharm.* 1999;56(1):57-62.
19. Embrey D. SHERPA: A systematic human error reduction and prediction approach. Paper presented at: International Topical Meeting Advances in Human Factors in

For peer review only

Nuclear Power Systems1986; American Nuclear Society, La Grange Park, Knoxville Tennessee, IL.

20. McLeod MC, Barber N, Franklin BD. Methodological variations and their effects on reported medication administration error rates. *BMJ Qual Saf.* 2013;22(4):278-289.
21. Lisby M, Nielsen LP, Brock B, Mainz J. How are medication errors defined? A systematic literature review of definitions and characteristics. *Int J Qual Health Care.* 2010;22(6):507-518.
22. Kozer E, Scolnik D, Macpherson A, et al. Variables associated with medication errors in pediatric emergency medicine. *Pediatrics.* 2002;110(4):737-742.
23. Jain S, Basu S, Parmar VR. Medication errors in neonates admitted in intensive care unit and emergency department. *Indian J Med Sci.* 2009;63(4):145-151.
24. Porter E, Barcega B, Kim TY. Analysis of medication errors in simulated pediatric resuscitation by residents. *West J Emerg Med.* 2014;15(4):486-490.
25. Service CsAT. Electronic Drug Chart for Referring Hospitals. http://site.cats.nhs.uk/wp-content/uploads/2014/11/dgh_drugcalculator_v1.2.pdf.
26. Potts MJ, Phelan KW. Deficiencies in calculation and applied mathematics skills in pediatrics among primary care interns. *Arch Pediatr Adolesc Med.* 1996;150(7):748-752.
27. Fineberg SL, Arendts G. Comparison of two methods of pediatric resuscitation and critical care management. *Ann Emerg Med.* 2008;52(1):35-40 e13.
28. Campagne DD, Young M, Wheeler J, Stroh G. Pediatric Tape: Accuracy and Medication Delivery in the National Park Service. *West J Emerg Med.* 2015;16(5):665-670.
29. Damhoff HN, Kuhn RJ, Baker-Justice SN. Medication preparation in pediatric emergencies: comparison of a web-based, standard-dose, bar code-enabled system and a traditional approach. *J.* 2014;19(3):174-181.

For peer review only - http://bmjopen.bmj.com/site/about/guidelines.xhtml

Figure 1. Overview of study objectives and analyses

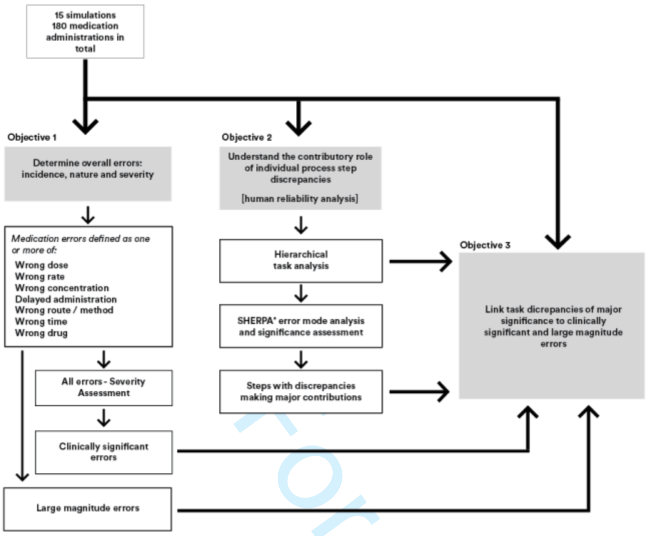

*SHERPA = systematic human error reduction and prediction approach

For peer review only

Figure 2. Significance-weighted heat-map of error by process step and SHERPA error mode

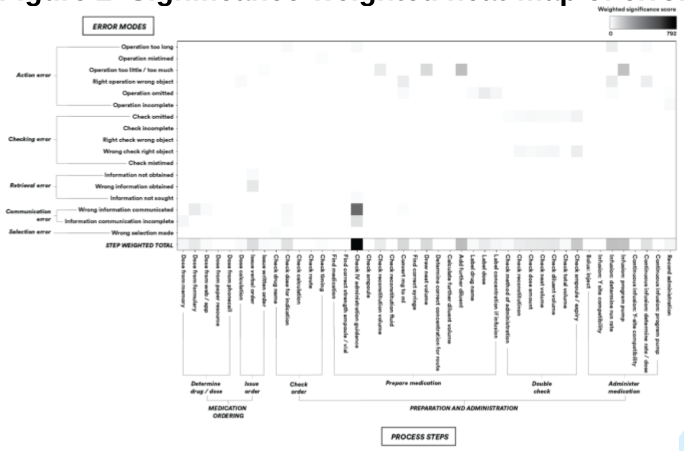

Figure 3: The hierarchical task analysis

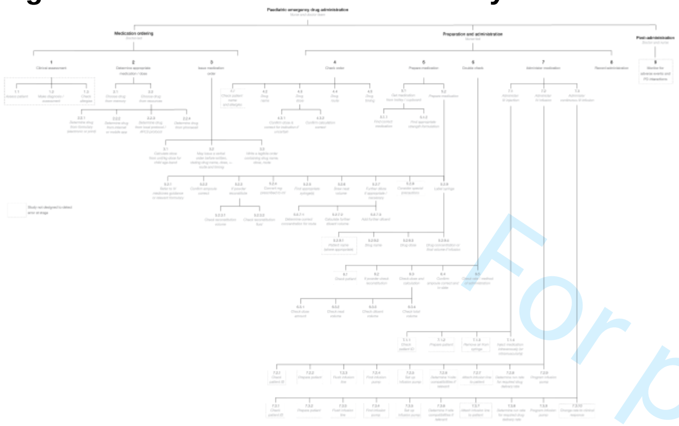

For peer review only

Appendices

Appendix 1. Case vignettes, expected medications and acceptable dose ranges

Case 1: Fitting Child

An 8-month-old, 8kg, previously well infant presents in prolonged status epilepticus with a two day history of coryza and one day history of lethargy and fever. On assessment the infant is having a generalised tonic clonic seizure and is febrile.

Teams were expected to treat the child for status epilepticus as per the standard paediatric guidelines, as well as add appropriate antimicrobial cover for presumed meningo-encephalitis. Additionally, all electrolyte abnormalities, including hypoglycaemia (glucose level initially 2.2), should be corrected as they would contribute to seizure activity.

In this case the following drugs were expected to be administered intravenously (in order of clinical priority).

- Lorazepam two doses (0.1mg/kg)
- 10% dextrose for hypoglycaemia (2-5ml/kg) [higher dose accepted as per British National Formulary]
- Phenytoin, loading dose (20mg/kg)
- Rapid Sequence Induction
    - Thiopentone (2-4mg/kg)
    - Suxamethonium (2mg/kg)
- Ceftriaxone (80-100mg/kg)
- Aciclovir (500mg/m^2 = 26mg/kg in this case)
- Clarithromycin (7.5mg/kg)
- Sedation and paralysis post intubation
    - Morphine infusion (10-40mcg/kg/hour)
    - Midazolam infusion (1-6mcg/kg/min)
    - Vecuronium infusion (0.8-3mcg/kg/min)

For peer review only

Case: Sepsis with shock

A 10-month-old, 9kg, previously well and fully immunised infant presents with shock and presumed meningococcal septicaemia. The infant has a two-day history of cough, coryza and fever, and has developed a spreading rash all over his body. On assessment the infant is floppy, only responsive to pain, with a spreading non-blanching purpuric rash all over his torso. He is febrile (40°C) with cold extremities and extremely poor perfusion. He has a sinus tachycardia with an unrecordable non-invasive blood pressure.

Teams were expected to initiate aggressive management of shock with fluids and vasoactive drugs in order to stabilise the infant. Early administration of appropriate antimicrobials was expected, as was the correction of metabolic derangements are also required.

In this case this would mean the following drugs should be administered (in order of clinical priority). Intraosseous access was expected to be established prior to successful intravenous cannulation.

- Ceftriaxone (80-100mg/kg)
- 10% dextrose for hypoglycaemia (2-5ml/kg) [higher dose accepted as per British National Formulary]
- Fluid resuscitation (fluid boluses were not measured for this study)
- Adrenaline infusion (0.1-0.5mcg/kg/min)
    - or
- Dopamine infusion (5-20mcg/kg/min) + adrenaline infusion
- Calcium for hypocalcaemia (0.11mmol/kg)
- Sodium Bicarbonate for acute mixed metabolic/respiratory acidosis (1ml/kg of 8.4%)
- Rapid Sequence Intubation
    - Ketamine (1-2mg/kg)
    - Suxamethonium (2mg/kg)
- Sedation and paralysis post intubation
    - Morphine infusion (10-40mcg/kg/hour)

For peer review only

- Midazolam infusion (1-6mcg/kg/min)
- Vecuronium infusion (0.8-6mcg/kg/min)

For peer review only

Appendix 2. Detailed operational definitions and explanation

Main outcome measures and definitions of error

Medication errors

- **Administrations were classed as <u>dosing errors</u> where:**
  - For drugs given by **injection** (bolus), there was a 10% or greater deviation from the recommended dosing range (DRDR). The DRDR was calculated as the absolute value of the percentage deviation from the recommended dose or dose range.
  - For medications administered as **infusions**, there was a 10% or greater deviation in either the DRDR or the deviation from the recommended dosing rate (DRDRate). The DRDRate was calculated as the absolute value of the percentage deviation from the recommended rate of administration for each medication.
  - For medications administered as **continuous infusions**, there was a 10% or greater deviation from the DRDRate.

- **<u>Timing errors</u>**
  - There is no study to describing acceptable times to medication administration for individual drugs in paediatric emergencies that it is possible to use as a performance benchmark. This lack of clarity is further complicated by the fact that after the successful preparation of a medication to be administered, it is regularly the case that practical issues such as line availability introduce delays. To make an assessment of prolonged administrations, therefore, times to drug 'ready for delivery' (tDRD) were used as a point of reference. The tDRD was calculated as the sum of the elapsed times during which the doctors were seeking medication information in resources with the total nurse-led preparation time. The timings used in this study are shown in more detail in the accompanying figure below.

For peer review only

- tDRD's were considered prolonged where a particular team took greater than 200% of the median tDRD for that specific drug across the entire study sample.
- Drugs given at the wrong time were classed as *'Wrong time'* errors
- Overall delays in tDRD due to clinical reasons as determined by observation, for example interruptions in order to perform critical tasks or give more important medications, were excluded as errors.

Figure 1. Timing definitions used for overall medication errors and individual process steps

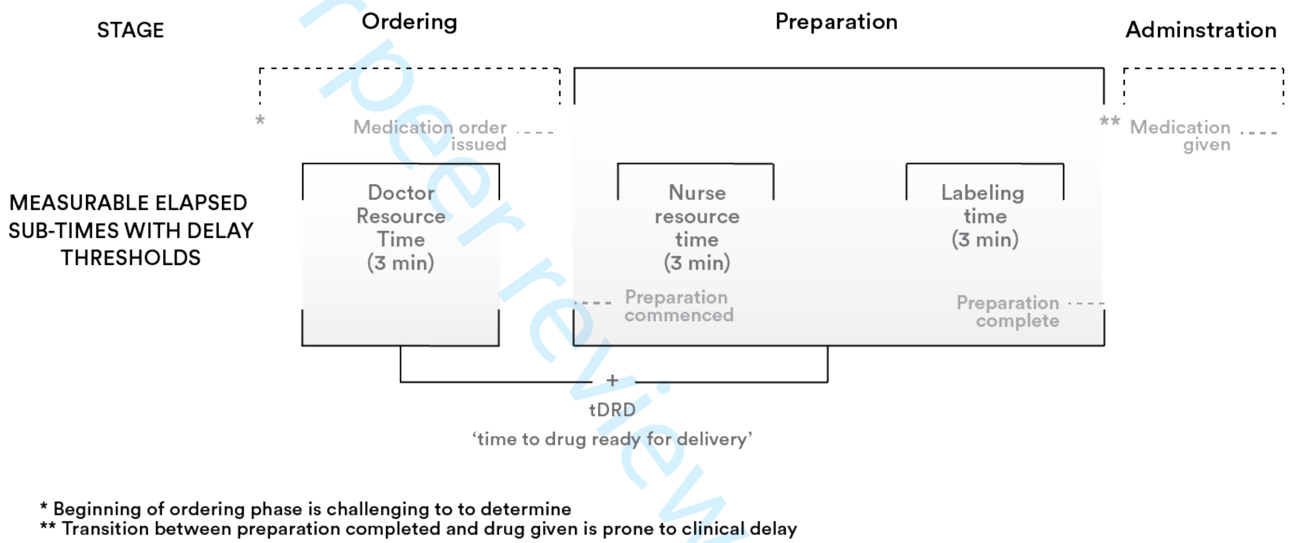

- **Other errors**
  - In addition, medication administrations were considered erroneous overall where
  - There was deviation from the recommended concentration (DRC) of greater than 10% from the suggested concentration in the IV medicines guidance.
  - There was a wrong drug error, a deviation from the suggested route or method of administration.

For peer review only

- All administrations **considered erroneous overall** were presented for severity assessment.

At the level of the task: the HRA / SHERPA framework

1. Task analysis

A hierarchical task analysis (HTA) of the procedure for the administration of bolus as well as medications administered by infusion was undertaken, adapting the Lane framework [3] for local emergency practice. The task analysis divided the procedure into three 'doctor-led' and five 'nurse-led' parent tasks, and each of these was broken down hierarchically into exhaustive sub-tasks. The HTA was assessed for face validity by five senior nurses in the study hospital.

2. Error mode categorisation and assessment

To understand the steps that were prone to introducing error, we used a generic human error taxonomy based on the SHERPA External Error Modes [4], with the addition of one additional error mode. Each step discrepancy was coded against this framework.

- **Coding thresholds for specific steps**
  - Step discrepancies that **made a contribution of more than 5% DRDR or DRDRate to an overall administration were coded as erroneous**. The lower threshold was chosen to identify causes for errors that were the result of cumulative step discrepancies.
  - There is no guidance with respect to acceptable times for retrieval of medication information from print or electronic resources. Guided by the expert panel, it was decided to consider times for either doctors or the nursing team to retrieve the appropriate information prolonged where they took more than 3 mins at any individual resource.
  - The following were considered the minimum requirements for safe labelling practice:
    - For IV injections: drug name and dose

For peer review only

- For IV infusions and continuous infusions: drug name, dose and strength of prepared mixture after dilution.

Where there were more than two discrepancies at a single step for a specific administration, the nurse evaluator made a subjective assessment of which error had the greater overall consequence and assigned an error mode to that discrepancy only.

3. Downstream or dependent errors

Root cause analysis (RCA) is a technique used to attempt to establish how and why an incident occurred [5]. While this study was not a typical RCA, to capture only 'root-cause' system vulnerabilities, steps where an action was performed correctly, but which perpetuated a previous error were not classed as erroneous. For example, a drug may be prescribed incorrectly, but then drawn up correctly with respect to the prescription. In this case, a prescription error would be recorded, while the drawing up step would not be classed as erroneous. Other errors which are independent of the prescription error, for example failure to double check the medication, were coded as discrepancies if present.

4. Significance assessment for individual step discrepancies

Medication preparation and administration is regularly subject to many minor sub-step discrepancies which may not individually, or even in combination, result in clinically significant error reaching the patient. In order to understand those sub-task errors which contributed to errors overall, significance scores were attributed to each coded sub-step error as follows:

- **No contribution**: discrepancy made no contribution to overall error
- **Minor contribution**: some contribution to overall error. A 5% threshold (for DRDR, DRDRate, DRC) was used to detect task discrepancies contributing in combination to significant errors.
- **Major contribution**: a principally contributory sub-task discrepancy that led directly to the medication error

For peer review only

References

1. Garfield, S., et al., *Measuring the severity of prescribing errors: a systematic review.* Drug Saf, 2013. **36**(12): p. 1151-7.
2. Dean, B.S. and N.D. Barber, *A validated, reliable method of scoring the severity of medication errors.* Am J Health Syst Pharm, 1999. **56**(1): p. 57-62.
3. Lane, R., N.A. Stanton, and D. Harrison, *Applying hierarchical task analysis to medication administration errors.* Applied ergonomics, 2006. **37**(5): p. 669-79.
4. Embrey, D. *SHERPA: A systematic human error reduction and prediction approach*. in *International Topical Meeting Advances in Human Factors in Nuclear Power Systems*. 1986. American Nuclear Society, La Grange Park, Knoxville Tennessee, IL.
5. United States Department of Veteran Affairs., *Root cause analysis*. VA National Center for Patient Safey, 2015. http://www.patientsafety.va.gov/professionals/onthejob/rca.asp

For peer review only

STROBE Statement

	Item No	Recommendation
**Title and abstract**	1	(*a*) Indicate the study's design with a commonly used term in the title or the abstract **[Title, abstract and methods section of manuscript]**
		(*b*) Provide in the abstract an informative and balanced summary of what was done and what was found **[See results section of abstract]**
**Introduction**		
Background/rationale	2	Explain the scientific background and rationale for the investigation being reported **[page 2]**
Objectives	3	State specific objectives, including any prespecified hypotheses **[page 2]**
**Methods**		
Study design	4	Present key elements of study design early in the paper **[pages 3 and 4]**
Setting	5	Describe the setting, locations, and relevant dates, including periods of recruitment, exposure, follow-up, and data collection **[page 3]**
Participants	6	(*a*) Give the eligibility criteria, and the sources and methods of selection of participants. Describe methods of follow-up **[page 3]**
		(*b*) For matched studies, give matching criteria and number of exposed and unexposed **[N/A]**
Variables	7	Clearly define all outcomes, exposures, predictors, potential confounders, and effect modifiers. Give diagnostic criteria, if applicable **[pages 4 and 5]**
Data sources/ measurement	8*	For each variable of interest, give sources of data and details of methods of assessment (measurement). Describe comparability of assessment methods if there is more than one group **[page 4]**
Bias	9	Describe any efforts to address potential sources of bias **[page 4]**
Study size	10	Explain how the study size was arrived at **[pages 3 and 8]**
Quantitative variables	11	Explain how quantitative variables were handled in the analyses. If applicable, describe which groupings were chosen and why **[pages 6 and 7]**
Statistical methods	12	(*a*) Describe all statistical methods, including those used to control for confounding **[page 6 and 7]**
		(*b*) Describe any methods used to examine subgroups and interactions **[page 6 and 7]**
		(*c*) Explain how missing data were addressed **[N/A]**
		(*d*) If applicable, explain how loss to follow-up was addressed **[N/A]**
		(*e*) Describe any sensitivity analyses **[N/A]**
**Results**		
Participants	13*	(a) Report numbers of individuals at each stage of study—eg numbers potentially eligible, examined for eligibility, confirmed eligible, included in the study, completing follow-up, and analysed **[page 7 and table 1]**
		(b) Give reasons for non-participation at each stage **[N/A]**
		(c) Consider use of a flow diagram **[N/A – described in text]**
Descriptive data	14*	(a) Give characteristics of study participants (eg demographic, clinical, social) and information on exposures and potential confounders **[page 7 and 8 and table 1]**
		(b) Indicate number of participants with missing data for each variable of interest **[table 1]**
		(c) Summarise follow-up time (eg, average and total amount) **[N/A]**
Outcome data	15*	Report numbers of outcome events or summary measures over time **[N/A]**
Main results	16	(*a*) Give unadjusted estimates and, if applicable, confounder-adjusted estimates and

For peer review only - http://bmjopen.bmj.com/site/about/guidelines.xhtml

		their precision (eg, 95% confidence interval). Make clear which confounders were adjusted for and why they were included **[N/A]**
		(*b*) Report category boundaries when continuous variables were categorized **[pages 3 and 4, tables 2 and 3]**
		(*c*) If relevant, consider translating estimates of relative risk into absolute risk for a meaningful time period **[N/A]**
Other analyses	17	Report other analyses done—eg analyses of subgroups and interactions, and sensitivity analyses **[pages 8, 9, 10]**
**Discussion**		
Key results	18	Summarise key results with reference to study objectives **[page 14]**
Limitations	19	Discuss limitations of the study, taking into account sources of potential bias or imprecision. Discuss both direction and magnitude of any potential bias **[page 15]**
Interpretation	20	Give a cautious overall interpretation of results considering objectives, limitations, multiplicity of analyses, results from similar studies, and other relevant evidence **[page 15 and 16]**
Generalisability	21	Discuss the generalisability (external validity) of the study results **[page 14]**
**Other information**		
Funding	22	Give the source of funding and the role of the funders for the present study and, if applicable, for the original study on which the present article is based **[within acknowledgements on title page]**

*Give information separately for exposed and unexposed groups.

Note: An Explanation and Elaboration article discusses each checklist item and gives methodological background and published examples of transparent reporting. The STROBE checklist is best used in conjunction with this article (freely available on the Web sites of PLoS Medicine at http://www.plosmedicine.org/, Annals of Internal Medicine at http://www.annals.org/, and Epidemiology at http://www.epidem.com/). Information on the STROBE Initiative is available at http://www.strobe-statement.org.

For peer review only

For peer review only - http://bmjopen.bmj.com/site/about/guidelines.xhtml

BMJ Open

Medication errors during simulated paediatric resuscitations: a prospective, observational human reliability analysis

Journal:	*BMJ Open*
Manuscript ID	bmjopen-2019-032686.R1
Article Type:	Original research
Date Submitted by the Author:	23-Sep-2019
Complete List of Authors:	Appelbaum, Nicholas; Imperial College London, Department of Surgery and Cancer, Division of Surgery; Imperial College London, NIHR-Imperial Patient Safety Translational Research Centre<br>Clarke, Jonathan; Imperial College London, NIHR-Imperial Patient Safety Translational Research Centre; Imperial College London, Centre for Mathematics of Precision Healthcare<br>Feather, Calandra; Imperial College London, Department of Surgery and Cancer, Division of Surgery; Imperial College London, NIHR-Imperial Patient Safety Translational Research Centre<br>Franklin, Bryony; Imperial College London, NIHR-Imperial Patient Safety Translational Research Centre; University College London School of Pharmacy<br>Sinha, Ruchi; Imperial College Healthcare NHS Trust, Department of Paediatric Intensive Care, Division of Women and Children's Services<br>Pratt, Phillip; Imperial College London Helix Centre for Design in Healthcare<br>Maconochie, Ian; Imperial College London, Department of Emergency Medicine, Division of Medicine<br>Darzi, Ara; Imperial College London, Department of Surgery and Cancer, Division of Surgery; Imperial College London, NIHR-Imperial Patient Safety Translational Research Centre
**Primary Subject Heading**:	Paediatrics
Secondary Subject Heading:	Emergency medicine, Intensive care, Nursing
Keywords:	PAEDIATRICS, Paediatric A&E and ambulatory care < PAEDIATRICS, Paediatric anaesthesia < PAEDIATRICS

SCHOLARONE™
Manuscripts

Medication errors during simulated paediatric resuscitations: a prospective, observational human reliability analysis

Nicholas Appelbaum (1, 2, 3), Jonathan Clarke (2, 3, 8), Calandra Feather (1, 2, 3), Bryony Dean Franklin (3, 4, 5), Ruchi Sinha (6), Philip Pratt (2), Ian Maconochie (7) and Ara Darzi (1, 2, 3)

Author Affiliations:

1 – Department of Surgery and Cancer, Division of Surgery, Imperial College London, UK

2 – Helix Centre for Design in Healthcare, Imperial College London, UK

3 - NIHR-Imperial Patient Safety Translational Research Centre, Imperial College London, UK

4 –UCL School of Pharmacy, University College London

5 – Centre for Medication Safety and Service Quality (CMSSQ), Imperial College Healthcare NHS Trust, London, UK

6 – Department of Paediatric Intensive Care, Division of Women and Children's Services, Imperial College London NHS Trust, UK

7 – Department of Emergency Medicine, Division of Medicine, Imperial College London, UK

8 - Centre for Mathematics of Precision Healthcare, Imperial College London

For peer review only

Keywords:
Paediatrics; Paediatric resuscitation, and paediatric ambulatory care; Paediatric anaesthesia

Word counts:
Abstract: 257 words
Manuscript: 3929 words

Author contributions:
NA, CF, JC and RS designed the study. RS facilitated the simulations, with NA and CF observing and coordinating. CF analysed the video footage. NA and JC were responsible for the data analysis which was reviewed by PP. BDF and IM advised on error definitions and analysis. NA drafted the study report, which was reviewed by all authors. All authors read and approved the final draft.

Full contact information for corresponding author:

Calandra Feather
Department of Surgery and Cancer
Division of Surgery
Imperial College London
10th Floor, QEQM Building
St Mary's Hospital
Praed Street, London, W2 1NY

Mob: +44 7977185577

Email: c.feather@imperial.ac.uk

Web: www.imperial.ac.uk/ighi
www.helixcentre.com

For peer review only

Medication errors during simulated paediatric resuscitations: a prospective, observational human reliability analysis

Abstract

Introduction: Medication errors during paediatric resuscitation are thought to be common. However, there is little evidence about the individual process steps that contribute to such medication errors in this context.

Objectives: To describe the incidence, nature and severity of medication errors in simulated paediatric resuscitations, and to employ human reliability analysis to understand the contribution of discrepancies in individual process steps to the occurrence of these errors.

Methods: We conducted a prospective observational study of simulated resuscitations subjected to video micro-analysis, identification of medication errors, severity assessment and human reliability analysis in a large English teaching hospital. Fifteen resuscitation teams of two doctors and two nurses each conducted one of two simulated paediatric resuscitation scenarios.

Results: At least one medication error was observed in every simulated case, and a large magnitude (>25% discrepant) or clinically significant error in 11 of 15 cases. Medication errors were observed in 29% of 180 simulated medication administrations, 40% of which considered to be moderate or severe. These errors were the result of 884 observed discrepancies at a number of steps in the drug ordering, preparation and administration stages of medication use, 8% of which made a major contribution to a resultant medication error. Most errors were introduced by discrepancies during drug preparation and administration.

Conclusions: Medication errors were common with a considerable proportion likely to result in patient harm. There is an urgent need to optimise existing systems and to commission research into new approaches to increase the reliability of human interactions during administration of medication in the paediatric emergency setting.

For peer review only

For peer review only - http://bmjopen.bmj.com/site/about/guidelines.xhtml

Strengths and limitations of this study

- This study adds to the literature base, highlighting paediatric emergency medication error as worrying, potentially highly harmful and requiring urgent attention.
- This study is one of the first to use Human Reliability Assessment to link task discrepancies with resultant medication errors, as well as to link these discrepancies directly to potential harm. This effort has demonstrated that a significant fraction of the burden of error in the paediatric emergency drug administration process originates during the preparation and administration phase and that most of these errors are likely to be undetected in clinical practice.
- Although we went to considerable lengths to replicate the paediatric emergency environment, the simulation environment cannot truly reflect the clinical environment during a genuine emergency.
- Furthermore, this study was conducted at a single site and participants were not blinded to the purpose of the study, so it is potentially subject to preparation bias.
- Participants were recruited from the paediatric emergency unit, intensive care unit and general paediatrics ward and had variable experience of emergency cases. However, all participants worked in clinical units that manage critically ill children.

For peer review only

Introduction

Background

Medication errors are among the leading cause of avoidable harm in healthcare worldwide [1] and up to three times more common in children than in adults.[2] The paediatric emergency environment, characterised by urgency and fraught with interruptions, is one of the clinical areas most vulnerable to error. Medication administration in emergencies is complex as it requires successful interactions between different teams of medical and nursing staff, as well as between individual

For peer review only - http://bmjopen.bmj.com/site/about/guidelines.xhtml

members of these teams. An additional challenge relates to obtaining relevant medication information and translating this into the required dose and concentration of the correct drug to be administered by the correct route for the clinical indication, all in a necessarily short space of time. Medication errors in general, and medication administration errors in particular, are both under-detected and under-reported,[3] such that little is known of their incidence or impact during resuscitation. However, medication errors have been reported in 7 out of 10 simulated paediatric resuscitations,[4] with other recent simulation studies suggesting 26% [5] to 70% [6] of administered medicines being given at the wrong dose. Laboratory studies analysing syringes prepared for anaesthetic use have found at least 15% to be greater than 20% discrepant from the intended drug concentration.[7]

The broader, systems view of medical error, heralded by the Institute of Medicine's "To Err is Human" report, saw the widespread adoption of Reason's organisational accident model [8] in healthcare. More recently, human reliability analysis (HRA) techniques, previously commonplace only in other high risk industries, have become increasingly used in healthcare research.[9,10] HRA is based on the understanding that neither humans nor systems can be error-proof, and asserts that to improve safety and reliability, a thorough analysis of system vulnerabilities at a task level is needed, taking into account human-human and human-machine interactions.[11,12] Medication safety researchers have previously used an HRA technique, the systematic human error reduction and prediction approach (SHERPA), to identify system vulnerabilities in ward-based medication administration,[13] anaesthesia[14] and general surgery.[15] This approach, however, has not been used quantitatively in medication safety research and has not been applied to paediatric resuscitation.

Our objectives were to describe the incidence, nature and severity of medication errors in simulated paediatric resuscitations, and then use HRA to understand the contributory role played by individual process step discrepancies with a focus on those discrepancies contributing to large magnitude and/or clinically significant errors.

Methods

Study design and setting

For peer review only - http://bmjopen.bmj.com/site/about/guidelines.xhtml

This prospective observational study was conducted April to November 2017 in a medical simulation facility within a large teaching hospital. The hospital has a paediatric emergency department (seeing 27,000 children each year) and a comprehensive paediatric inpatient service (admitting 5,000 each year). The hospital used electronic prescribing in the inpatient setting, but during resuscitations, medications were more commonly ordered on paper prescription charts. We recruited resuscitation teams of four clinicians, that were randomised to participate in one of two standardised simulated paediatric resuscitation scenarios. The study was approved by the Health Research Authority and the hospital concerned. NHS ethics approval was not required. Participants gave written informed consent.

Patient and Public Involvement

The research team held a workshop with parents to get their feedback on the proposal, develop the PPIE plans, and identify future areas for research. We actively sought attendees through INVOLVE's "People in Research" website, social media and Imperial College London's existing networks. Our team has also participated in a PPIE event run by the Royal College of Paediatrics and Child Health, in collaboration with MedsIQ (http://www.medsiq.org) and Medicines for Children (http://www.medicinesforchildren.org.uk), two UK-based paediatric medication safety initiatives.

Participants

Eligible participants were a convenience sample of medical and nursing staff from the departments of paediatrics and paediatric emergency medicine at the study hospital. Participants were assigned into teams comprising a senior doctor (a specialist registrar, with at least a year of prior experience as a registrar), a junior doctor, a senior nurse (with at least 5 years' nursing experience) and a junior nurse.

Clinical scenarios

The two scenarios were:

A. Prolonged status epilepticus in an 8-month-old, 8kg child
B. Presumed meningococcal sepsis in a 10-month-old, 9kg child

For peer review only - http://bmjopen.bmj.com/site/about/guidelines.xhtml

The two scenarios (**Appendix 1)** were designed by a collaboration of paediatric nurses, emergency physicians, intensivists, general paediatricians, and anaesthetists. Face validity was established by an independent expert panel of six, with representation from each of these professional groups, including two lead paediatric clinical nurse educators. It was deemed that the two scenarios were both similarly demanding and clinically sound, with treatment recommendations corresponding closely to UK Resuscitation Council and Royal College of Paediatrics and Child Health teaching cases.

A simulated paediatric resuscitation bay was created. The mannequin used was a SimBaby Version 2 (Laerdal Medical, Stavanger, Norway), and the syringe pump stack consisted of Alaris PK MK4 units (Becton Dickinson, Franklin Lakes, USA). All relevant print materials (e.g. British National Formulary for Children [16] and local guidelines) and hospital information technology systems as well as external internet access were available. Participants were requested to prescribe, prepare and administer medications exactly as per usual practice, to use mobile applications or websites as they would in clinical practice, and to telephone specialist colleagues if required. A paediatric intensivist, the hospital lead for paediatric simulation, ran the scenarios. She provided standardised clinical information as live feedback and answered questions regarding the child's response to treatment or their current condition when needed.

Data sources and measurement

A Scotia Medical Observation System (Smots, Scotia UK, Edinburgh, UK), with two 3-axis, ceiling-mounted video cameras, and three mobile, high-definition cameras equipped with boom microphones, was used. Both nurses in each team wore head-mounted high-definition video cameras (GoPro Inc, California, USA). The video recordings were analysed by a research nurse with ten years' experience in paediatric intensive care.

Outcome measures

We reserved the term 'medication error' to describe an overall error with respect to a particular drug administration as a whole, after having been administered to a patient,

For peer review only - http://bmjopen.bmj.com/site/about/guidelines.xhtml

and the term 'discrepancy' to refer to observed deviations from expected local practice at the level of the individual task. This approach has been successfully used by other medication safety researchers.[17]

Variation in clinician practice and human adaption to the complex process of medication ordering, preparation and administration typically results in many minor task discrepancies that may not individually, or even in combination, result in a medication error or patient harm.[17] Other discrepancies almost always result in medication errors. For example, a prescribing or pump-programming discrepancy is highly likely to result in a medication error. To identify the most important task discrepancies, we assessed all observed discrepancies to establish the extent to which they may or may not have contributed to any resultant medication errors.

Figure 1 summarises the study objectives and associated analyses.

Medication errors

Medication errors included any errors in dose, administration rate, concentration, drug, route of administration, method of administration, timing, or delay in administration. Operational definitions for each of these are given in **Appendix 2.** Briefly, dosing errors were defined as a greater than 10% deviation from the recommended dosing range (DRDR) [18] at the study site. Any deviation from the recommended rate of administration (DRDRate) was calculated in a similar manner and deviations of more than 10% were considered to be medication errors. Where there was a greater than 25% discrepancy in the DRDR or DRDRate, the errors were considered as 'large magnitude'. Deviations from the recommended concentration (DRC) of greater than 10% from the concentration specified in local guidance were also included as medication errors.

To identify delayed administrations, the time taken for the dose to be 'ready for delivery' was calculated as the time for the doctors to obtain any medication information required plus the nurse-led preparation time. The time to be ready for deliver was considered 'prolonged' when a particular team took more than double the

For peer review only

median time for that specific drug across the entire study without clinical cause for the delay as determined by the nurse assessor. For example, if a medication administration was interrupted to reassess the patient clinically or to administer another medication as a priority, a prolonged time would be excluded as an error on clinical grounds.

Severity assessment

There are few validated tools that can be used to assess the potential severity of medication errors without knowledge of patient outcomes and that are thus usable in simulated studies [19]. One of these tools is that of Dean and Barber, based on 4-5 experts independently assessing each error on a 0 to 10 scale, and their mean score used as an index of severity. Mean scores under 3 suggest errors of minor severity, those between 3 and 7 as moderate and those greater than 7 as severe.[20] We used this approach, with two paediatric intensivists, one paediatric anaesthetist, one senior critical care nurse, and one senior clinical pharmacist assessing each error.

Discrepancies at the level of the task

A hierarchical task analysis (HTA) was developed based on a similar framework for ward-based medication administration[13] and assessed for face validity by five senior nurses in the study hospital. A generic human error taxonomy, based on the SHERPA external error modes[21] with one additional error mode, 'information not sought', was used to code observed discrepancies against the HTA.

Where there were more than two discrepancies at a single step for a specific administration, the nurse assessor made a subjective assessment of which had the greater overall consequence and assigned an error mode (Figure 2) to that discrepancy only. To capture 'root-cause' system vulnerabilities, steps where an action was performed correctly, but which perpetuated a previous medication error, were not classed as discrepancies. An example would be a correct volume calculation based on an incorrectly prescribed dose. In this example, the volume calculation which persisted but did not directly cause the error wouldn't be classed as a discrepancy whereas the incorrect prescription would be.

For peer review only - http://bmjopen.bmj.com/site/about/guidelines.xhtml

Significance assessment of task discrepancies

All task discrepancies were classified by the nurse assessor according to the contribution made by the discrepancy as follows:

No contribution: the discrepancy did not contribute to a medication error

Minor contribution: some contribution made to a medication error

Major contribution: the task discrepancy led directly to a medication error

For peer review only

Data management and analysis

Medication errors were described according to the type of error, method of administration (e.g. injection, infusion, continuous infusion) and stage of the medication use process (e.g. medication ordering, preparation, administration) during which the error occurred. Error rates were calculated using the total number of applicable administrations as the denominator.

Step discrepancies were presented as counts, grouped by task and contribution to medication error. Discrepancy rates were calculated as the percentage of discrepancies that made a major, minor or no contribution to an error, with the number of observed discrepancies at each process step as the denominator. Of those discrepancies making major contributions to medication errors, the proportion that led to clinically significant errors (severity score > 3) and/or large magnitude errors (DRDR or DRDRate > 25%) was also calculated.

There is no literature that quantifies the extent to which a step discrepancy having a minor or major contribution to an error is of greater significance than a step discrepancy that makes no contribution to an error. For a weighted, 'heat map' HRA analysis, it was therefore necessary to attribute different weights to discrepancies that resulted in error to those that did not. Sub-step discrepancies were therefore weighted, agreed by the expert panel, as follows:

- No contribution: weight = 1
- Minor contribution: weight = 10

- Major contribution: weight = 40

The total weighted significance score for each step was thereby calculated for each error mode.

Inter-observer reliability

One of the 15 simulations was re-analysed by an additional independent nurse not involved in the simulations. Spearman's rank correlation coefficient was calculated for 38 continuous variables (e.g. doses, elapsed times) and Cohen's Kappa for 20 categorical variables (e.g. reconstitution fluid, parameters for labelling quality assessment). The dataset concerning the medication errors and timing parameters was treated separately from the dataset containing the parameters for the HRA (the task discrepancies).

Results

Data were collected during 15 simulations according to participant availability. Eight simulations were of the prolonged seizures case, and seven of the meningococcal sepsis case. Participants comprised 30 doctors and 30 nurses **(Table 1)**, each of whom completed one simulation. For categorical variables, Cohen's Kappa values were between 0.862 (medication error) and 0.954 (HRA). Continuous variables were only present in the medication error dataset, for which Spearman's rank coefficient was greater than 0.904 for all variables.

For peer review only

Table 1. Characteristics of study population

	Overall	Doctors	Nurses
**Total number of participants**	60	30	30
**Age †**			
Median (range)	30 (23-51)	30.5 (23-44)	28 (23-51)
**Gender (%)**			
Female	52 (87%)	23 (77%)	29 (97%)
Male	8 (13%)	7 (23%)	1 (3%)
**Years in clinical practice (n, %)**			
0-5	32 (53%)	11 (37%)	21 (70%)
6-10	20 (33%)	16 (53%)	4 (13%)
11-15	5 (8%)	3 (10%)	2 (7%)
16-20	1 (2%)	0 (0%)	1 (3%)
>20	2 (3%)	0 (0%)	2 (7%)
**Years in paediatric practice (n, %)**			
0-5	37 (62%)	16 (53%)	21 (70%)
6-10	16 (27%)	12 (40%)	4 (13%)
11-15	5 (8%)	2 (7%)	3 (10%)
16-20	1 (2%)	0 (0%)	1 (3%)
>20	1 (2%)	0 (0%)	1 (3%)

† Age data was omitted for five participants

Medication errors

Participants conducted 180 medication administrations. Overall, errors were observed reaching the patient for 52 drug administrations (29%) and at least once in every simulation. Of these errors, 30 (58%) were assessed as being of minor severity, 16 (31%) as moderate and 6 (12%) as severe. There were 27 large magnitude errors (52% of all errors), in which the DRDR / DRDRate was greater than 25%. Of all erroneous administrations that reached the patient, only two (4%) were noticed by staff after administration and therefore may have been reported in clinical practice. A detailed error analysis is provided in **Table 2** and description of the ten most severe errors in **Table 3**.

Hierarchical task analysis

The full HTA is shown as **Figure 3** and shows all steps assessed in the paediatric emergency drug administration process.

For peer review only

Errors and discrepancies by stage of medication use and process sub-step

Overall, 884 step discrepancies were observed, excluding dependent downstream discrepancies after an initial discrepancy. Of these 884 step discrepancies, 174 (20%) were linked to a medication error, with 70 (8%) assessed as making a major contribution to an error, 104 (12%) making a minor contribution, and 710 (80%) making no contribution.

Figure 2 shows the significance-weighted HRA data represented as a heat-map demonstrating the relative contributions of discrepancies at each step and by each error mode to medication errors. **Table 4** summarises the discrepancy counts per step as well as the percentage of both large magnitude and clinically significant errors with a major contribution made at each HTA step.

Errors and discrepancies during medication ordering

We observed 170 discrepancies during the ordering phase. Five of the 22 clinically significant medication errors were due to discrepancies during medication ordering, with three of these due to incorrect dose information retrieval from the British National Formulary for Children. The majority of the remaining discrepancies (136) were due to incomplete verbal medication orders based on which drug preparation commenced, two of which resulted in medication errors. Of the 180 written medication orders examined, there were six discrepancies observed, all of which were corrected by the clinicians and therefore made no contribution to any dosing errors, but did result in one delayed administration.

For peer review only

Table 2. Incidence, nature and severity of errors, presented by phase and type of error and method of administration

	Number of errors	Incidence as % of total administrations (n=180)	Incidence as % of administrations by method of administration	Severity assessment: Minor (severity score < 3) (n)	Severity assessment: Moderate (severity score 3 - 7) (n)	Severity assessment: Severe (severity score > 7) (n)	Severity assessment: Mean severity score
**Any error**	**52**	**29%**		**30**	**16**	**6**	**3.2**
By stage of medication use and error type							
**Prescription errors**	**8**	**4%**		**2**	**4**	**2**	**5.1**
Wrong dose	8	4%	-	2	4	2	5.1
Wrong route	1	1%	-	-	-	1	-
Wrong drug	-	-	-	-	-	-	-
**Preparation and administration errors**	**40**	**22%**		**25**	**12**	**5**	**3.1**
Wrong drug	1	1%	-	-	-	1	-
Wrong dose prepared	10	6%	-	6	3	1	2.8
Wrong diluent / concentration	20	11%	-	12	6	2	3.0
Wrong rate (infusions)	11	6%	-	4	5	2	4.2
Wrong route / method	9	5%	-	5	3	1	3.6
Wrong time	1	1%	-	-	-	1	-
By method of administration and error magnitude							
**Bolus doses (n = 77 in total)**							
**Any error**	**24**	**-**	**31%**	**13**	**7**	**4**	**3.6**
Dose error, DRDR† > 10%	7	-	9%	-	5	2	5.0
Dose error, DRDR† > 25%	6	-	8%	-	4	2	5.7
Delayed administration, dose correct	5	-	7%	2	2	1	4.2
Delayed administration, dose error	-	-	-	-	-	-	-
**Intermittent infusions (n = 48 in total)**							
**Any error**	**18**	**-**	**38%**	**10**	**7**	**1**	**3.2**
Total dose error, DRDR† > 10%	5	-	10%	3	2	-	3.7
Total dose error, DRDR †> 25%	3	-	6%	1	2	-	5.2
Rate error, DRDRate‡ > 10%*	8	-	17%	2	5	1	4.6
Rate error, DRDRate‡ > 25%*	6	-	13%	-	5	1	6.0
Delayed administration, correct dose and rate	2	-	4%	2	-	-	1.4
Delayed administration, incorrect dose or rate	1	-	2%	-	1	-	-
**Continuous infusions (n = 55 in total)**							
**Any error**	**10**	**-**	**18%**	**7**	**2**	**1**	**2.2**
Delivery rate error, DRDRate‡ > 10%	5	-	9%	2	2	1	3.4
Delivery rate error, DRDRate‡ > 50%	5	-	9%	2	2	1	3.4
Delayed administration, correct delivery rate	1	-	2%	1	-	-	-
Delayed administration, with incorrect delivery rate	1	-	2%	-	1	-	-

More than one error type can occur in one medication administration and a single medication error may meet more than one criterion, so individual error types do not sum to the total by stage or method. Other error types (for example diluent errors) are included in the 'any error' counts but are not presented as sub-counts.

*Rate errors are only shown for intermittent infusions where the delivery rate error is due to a pump-programming error.

† DRDR (deviation from recommended dosing range) = absolute value of the percentage difference from the recommended dose or dose range

‡ DRDRate (deviation from recommended dosing rate) = absolute value of the percentage difference from the recommended rate of administration

For peer review only

Table 3. Details of the ten most severe medication errors by severity score

Rank	Medication	Stage	DRDR †	DRDRate ‡	Other error	Severity score	Error detail	Error cause
1	Thiopentone	Ordering	900%	-	-	8.8	320mg rather than 32mg given as IV bolus	Doctors asked for two doses of thiopentone to be prepared which were ordered at 32mg each. Nurse read phenytoin dose, which was 160mg, written on the line above the thiopentone order on the medication chart. Two boluses of 160mg rather than 32mg were prepared and administered.
2	Calcium chloride	Ordering	789%	-	-	8.6	8.8mmol rather than 0.99mmol given as IV bolus	Incorrect dose for indication selected and prescribed from British National Formulary (BNF)
3	Thiopentone	Administration	-	-	Timing error	8.2	Given prior to anaesthetist ready	Medication prepared and administered prior to medical and anaesthetic team being ready to manage airway and breathing
4	Dextrose	Preparation	-	-	14 minutes taken to administer	7.5	825% of median time for dextrose. Hypoglycaemic patient, glucose = 2.2	Excessive time spent working out required volume to administer due to confusion caused when checking concentration information across multiple resources
5	Adrenaline	Preparation	-	-88.9%	18 minutes taken to administer	7.5	286% of median time for adrenaline infusions	Team unfamiliar with prescribing and preparing adrenaline infusion, time spent accessing multiple resources
6	Phenytoin	Preparation / administration	-	300%	-	7.2	Correct dose given undiluted at four times the recommended administration rate (4mg/kg/min rather than 1mg/kg/min)	Calculation error when setting up rate on pump, administered over 5 minutes instead of 20 minutes. Undiluted phenytoin infusions are not in accordance with local policy but this was not clearly indicated in the IV administration guidance
7	Phenytoin	Administration		-99.2%	-	6.9	Correct dose given too slowly (0.0078mg/kg/min rather than 1mg/kg/min)	Calculation error when setting up rate on pump
8	Aciclovir	Ordering and administraiton	-45.5%	163.3%	-	6.8	105mg rather than 210mg given as infusion and run over 20 mins instead of an hour	Incorrect dose chosen for indication from BNF and administered over 20 mins instead of 1 hour. Correct dose was on following page of BNF
9	Aciclovir	Ordering	-45.5%	-	-	6.2	105mg instead of 210mg	Incorrect dose selected for indication from BNF. Correct dose was on following page of BNF
10	Aciclovir	Preparation / administration	-	high	-	5.7	Given as a neat bolus rather than being diluted and infused	Administered as bolus instead of as an infusion over 1 hour

† DRDR (deviation from recommended dosing range) = percentage difference from the recommended dose or dose range

‡ DRDRate (deviation from recommended dosing rate) = percentage difference from the recommended rate of administration

For peer review only

For peer review only - http://bmjopen.bmj.com/site/about/guidelines.xhtml

Table 4. Number, frequency and relationship of discrepancies to resultant errors with sub-analysis of major discrepancies that resulted in large magnitude and clinically significant errors

		Relationship to resultant errors				
				Discrepancies which made a major contribution to a medication error		
Stage of deviation	Total discrepancies (n)	No contribution n (%*)	Minor contribution n (%*)	Major contribution, total (n, %*)	Discrepancies that resulted in clinically significant errors (n, %*) †	Discrepancies that resulted in large magnitude errors n (%*) ‡
**OVERALL**	**884**	**710 (80.3)**	**104 (11.8)**	**70 (7.9)**	**33 (47.1)**	**31 (44.3)**
**ORDERING PHASE**	**170**	**159 (93.5)**	**2 (1)**	**9 (5)**	**5 (23)**	**6 (22)**
**Determine dose**	**29**	**21 (72)**	**1 (3)**	**7 (24)**	**5 (23)**	**6 (22)**
Dose from memory	4	2 (50)	-	2 (50)	-	1 (4)
Dose from formulary	10	6 (60)	1 (10)	3 (30)	3 (14)	3 (11)
Dose from other resource	11	10 (91)	-	1 (9)	1 (5)	1 (4)
Dose calculation	4	3 (75)	-	1 (25)	1 (5)	1 (4)
**Issue order**	**141**	**138 (97.9)**	**1 (1)**	**2 (1)**	**-**	**-**
Issue verbal order	136	134 (98.5)	1 (1)	1 (1)	-	-
Issue written order	5	4 (80)	-	1 (20)	-	-
**PREPARATION PHASE**	**588**	**456 (77.6)**	**89**	**43 (7)**	**17 (77.3)**	**13 (48)**
**Check order**	**19**	**6 (32)**	**9 (47)**	**4 (21)**	**3 (14)**	**1 (4)**
Check drug name	1	-	-	1 (100)	1 (5)	1 (4)
Check dose for indication	15	5 (33)	8 (53)	2 (13)	1 (5)	-
Check calculation	1	-	1 (100)	-	-	-
Check route and timing	2	1 (50)	-	1 (50)	1 (5)	-
**Preparation, actual**	**310**	**263 (84.8)**	**8 (3)**	**39 (13)**	**14 (64)**	**12 (44.4)**
Find correct medication and strength of vial	2	-	2 (100)	-	-	-
Check intravenous administration guidance	42	22 (52)	1 (2)	19 (45)	9 (41)	6 (22)
Check ampoule	1	-	1 (100)	-	-	-
Check reconstitution fluid and volume	7	3 (43)	1 (14)	3 (43)	-	-
Convert milligrams to millilitres	7	2 (29)	-	5 (71)	3 (14)	4 (15)
Find correct syringe and draw neat volume	8	3 (38)	-	5 (63)	1 (5)	1 (4)
Determine correct concentration for route	2	2 (100)	-	-	-	-
Calculate further diluent volume and dilute	12	4 (33)	1 (8)	7 (58)	1 (5)	1 (4)
Label syringe with drug name, dose and concentration if appropriate	231	227 (98.3)	4 (2)	-	-	-
**Double checking**	**259**	**187 (72.2)**	**72 (27.8)**	**-**	**-**	**-**
Check reconstitution	9	5 (57)	4 (44)	-	-	-
Check dose and neat volume	69	48 (70)	21 (30)	-	-	-
Check diluent volume and total volume separately	86	63 (73)	23 (27)	-	-	-
Check ampoule + expiry	29	26 (90)	3 (10)	-	-	-
Check rate / method	66	45 (68)	21 (32)	-	-	-
**ADMINISTRATION PHASE**	**28**	**8 (29)**	**2 (7)**	**18**	**11 (50)**	**12 (44.4)**
Boluses: inject	2	1 (50)	1 (50)	-	-	-
Infusions: determine run rate	8	-	1 (13)	7 (88)	5 (23)	5 (19)
Determine Y-site compatibility	2	2 (100)	-	-	-	-
Determine delivery rate for continuous infusions	7	3 (43)	-	4 (57)	3 (14)	4 (15)
Program infusion pump	9	2 (22)	-	7 (78)	3 (14)	3 (11)
**RECORDING**	**88**	**88 (100)**	**-**	**-**	**-**	**-**

* The denominator for percentages is the total number of discrepancies in each row

† Number and percentage of clinically significant errors (severity score > 3) with major contributory discrepancies made at each specific step, of a total of 22 clinically significant errors.

‡ Number and percentage of large magnitude errors [deviation from recommended dosing range (DRDR) or deviation from recommended dosing rate (DRDRate) > 25%] with major contributory discrepancies made at each specific step, of a total of 27 large magnitude errors

For peer review only - http://bmjopen.bmj.com/site/about/guidelines.xhtml

Errors and discrepancies during medication preparation

During medication preparation, 310 discrepancies were observed, representing 35% of all observed discrepancies. These contributed to one medication error involving the wrong drug, ten dose errors and 20 diluent or dilution errors. The retrieval of preparation and administration information from the online IV medications guide was the step mostly likely to contribute to medication errors during medication preparation, with 42 discrepancies (19 major contribution to a medication error, one minor), resulting in nine clinically significant medication errors (severity score > 3). The retrieval of incorrect information and taking an excessively long time to identify the correct information within the guidance were the most common discrepancy error modes.

There were seven discrepancies when converting milligrams to millilitres of undiluted drug, and 18 discrepancies (six making a major contribution) when, after having made the correct calculations, nurses withdrew either the incorrect amount of undiluted drug or the incorrect amount of diluent into the syringe.

Double-checking

Overall, 259 discrepancies were observed during the double-checking phase, 72 (28%) of which made a minor contribution to a medication error. Checking the route and method (e.g. infusion or bolus) of administration was the most frequently omitted.

We observed 29 medication errors that were made during medication ordering and preparation but had not been yet administered to the patient at the point of double checking. These errors were thus potentially 'interceptable' but all ultimately reached the patient. Of these errors, in 14 cases, the double-checking interaction between the nurses included the incorrect step but failed to identify it as incorrect.

Errors and discrepancies during the administration phase

Of all observed discrepancies, only 28 (3%) occurred during administration. These resulted in 11 wrong rate errors (five moderate, two severe), nine wrong method errors

For peer review only - http://bmjopen.bmj.com/site/about/guidelines.xhtml

(three moderate, one severe) and one severe wrong time error. Discrepancies during the administration phase constituted a third of all discrepancies that made a major contribution to a clinically significant error.

Infusions in particular were prone to administration errors. Of the 17 discrepancies observed during infusion rate calculations or when programming the infusion pump for intermittent infusions, 14 were of major consequence, and accounted for 23% of all clinically severe errors. Seven discrepancies (four making a major contribution to a medication error) occurred when determining the delivery rate for continuous infusions.

Discussion

This prospective observational study is the first in paediatric emergency medicine to include a quantitative HRA, allowing identification of the task discrepancies making the greatest contribution to medication error. We identified at least one medication error in all 15 simulations, and a large magnitude or clinically significant error in 12 of these.

Comparison with previous literature

Historical heterogeneity of the definitions of medication error and the variability in reporting metrics make comparison with previous literature difficult.[22,23] Additionally, there are few simulated studies and no relevant clinical studies in paediatric resuscitation, making comparator data scarce.

Prescribing error rates in the (non-simulated) emergency setting have been reported to be between 10.1% and 16% [24,25] of all orders; our study reports a lower rate of 5%, although this difference may be at least partly due to different error definitions. Our study reinforces similar findings in another recent analysis [26] suggesting that preparation and administration errors may be more common, but is the first to highlight the extent to which these errors go undetected. Other simulated studies have reported error rates for the administration of intravenous bolus medication of between 15.5% and 26.5% [4,5,27]; in our study, it was 31%. The referenced studies, however, reported

For peer review only

only on dose errors, and not any other medication error types. Only seven out of the 24 medication errors we observed for bolus doses were dose errors. Medications given by intermittent infusion were the most error-prone in our study. There are no studies that investigate emergency administration of intermittent infusions in sufficient detail to provide a basis against which to compare this finding.

Medications given by continuous infusion are potentially the most complicated in paediatric emergencies. In addition to the preparation steps for intermittent infusions, staff generally have to convert infusion rates in milligrams or micrograms per kilogram per minute to infusion rates of millilitres per hour. A recent trial of a digital application reported errors in 70% of continuous vasopressor infusions [6] in the control arm. However, despite the increased cognitive demand, we observed the lowest incidence of medication error for continuous infusions, at 18%. Administration of continuous infusions in our hospital seemed to be relatively well supported by an online / paper tool [28].

Limitations

Although considerable efforts were made to replicate the paediatric ED environment in a state-of-the-art facility, the simulation environment may not reflect the clinical environment during a genuine emergency. Participants were not blinded to the purpose of the study, and therefore this investigation is potentially subject to preparation bias.

This investigation was conducted at a single site and all three of the nurse video assessors were from the same academic unit in the study hospital. It is thus possible that, despite best efforts at standardisation, the results may be skewed to reflect the expectations of medication practice at the study site.

Although this is one of the largest simulations studies in paediatric emergency medicine investigating medication errors, the sample size made it impossible to make probabilistic assessments of the relationship between step discrepancies and medication errors. Furthermore, the discrepancy assessment was made by a single nurse assessor. Save for the extract on which inter-rater reliability was tested, we did not have the resources to evaluate 884 discrepancies by more than one clinician, reaching consensus on each.

For peer review only - http://bmjopen.bmj.com/site/about/guidelines.xhtml

Finally, we did not investigate the potential role of clinical pharmacists in the resuscitation setting. There is convincing evidence that the presence of clinical pharmacists reduces medication errors in this setting [29-31], and the emergency department at the study site does, at times, benefit from the assistance and expertise of clinical pharmacists. However, their presence is not routine during resuscitations.

Implications for research and practice

This study highlights the need for research to optimise clinicians' use of electronic resources containing medication preparation and administration information. We were not able to pinpoint the precise steps at which the current electronic intravenous medications guidance system in the study hospital proved vulnerable to misinterpretation. Research to further understand the steps that need attention may serve as a useful basis from which to refine, and if needed, redesign such systems.

This study reaffirms that performing complex arithmetic in high-stress clinical environments is a considerable contributor to medication error.[32] With the purpose of addressing medication safety in paediatric resuscitation, the literature has been dominated by studies looking at 'resuscitation aids', most commonly length-based tapes.[18,33,34] These aids couple weight estimation with a suggested dose for a limited number of medications, but usually do not provide comprehensive preparation and administration support. It is not likely that length-based tapes would have decreased the rate of medication error in this study. Further clinical research is required to determine the effectiveness of new digital tools that do support preparation and administration, such as those that have shown promising results in simulated studies.[6,35]

Human factors methods have been used in other high-risk industries to define system vulnerabilities for building safer systems. By using quantitative HRA, this study provides evidence for the prioritisation of research efforts directed towards new interventions to address the most important system weaknesses.

In terms of implications for practice, one of the most unexpected findings in this investigation was the uncovering of 'purely mechanical' task discrepancies resulting

For peer review only - http://bmjopen.bmj.com/site/about/guidelines.xhtml

in medication errors. During drug preparation, clinicians were observed drawing up incorrect volumes of medications or diluents even though all calculations were correct. This suggests that efforts seeking to address medication safety in cognitively demanding environments using clinical education strategies or contemporary technologies must do so without disregarding the seemingly 'simplest' aspects of drug preparation. The reliability of information exchanges between healthcare professionals similarly needs improvement. Verbal medication orders in particular are inconsistent and error-ridden. Particular attention should be paid to medication orders given verbally in the emergency setting, using approaches such as the recipient verbally confirming the medication and dose being prepared. More importantly however, there is an urgent need for research to explore how to bring greater effectiveness to checking and double-checking more broadly. These are steps intended to defend patients from error, but which are too often ineffective.

Conclusions

Overall, we identified errors in 29% of all simulated medication administrations, only two of which were detected by participants, with 40% of these likely to result in moderate or severe harm. HRA revealed a number of error-prone steps, many of which occurred during preparation and administration of correctly ordered medications. The task most likely to result in erroneous medication administration was ineffective retrieval of correct medication preparation and administration instructions from intravenous medication guidance.

This study has highlighted an urgent need to optimise existing systems and to commission new approaches to increase the reliability of human interactions with the emergency medication administration process.

Acknowledgements

We would like to thank all clinical staff who participated in the simulations. It is our hope that the enthusiasm and extraordinary open-mindedness our dedicated and

For peer review only

deeply competent colleagues demonstrated in making this study possible will lead to safer systems and innovations in paediatric medication safety.

This study was funded by the NIHR-Imperial Patient Safety Translational Research Centre, with infrastructure support from the NIHR Imperial Biomedical Research Centre. Support was also received from the Resuscitation Council (UK).

Ethical approval: The Health Research Authority approved this study.

Contributorship

NA, CF, JC and RS designed the study. RS facilitated the simulations, with NA and CF observing and coordinating. AD provided funding. CF analysed the video footage. NA and JC were responsible for the data analysis which was reviewed by PP. BDF and IM advised on error definitions and analysis. NA drafted the study report, which was reviewed by all authors. All authors read and approved the final draft.

Competing interests

The Helix Centre at Imperial College London is leading an effort in collaboration with the British National Formulary developing digital tools in an attempt to improve paediatric medication safety. NA has written two patents describing syringe labelling techniques in medication safety. BDF supervises a PhD student part funded by a supplier of hospital electronic health record systems and has received funding from Pfizer for organising and chairing two symposia on medication safety.

We confirm that we have given due consideration to the protection of intellectual property associated with this work and that there are no impediments to publication, including the timing of publication, with respect to intellectual property. In so doing we confirm that we have followed the regulations of our institution concerning intellectual property.

Data availability statement

Non-identifiable data are available upon reasonable request. Raw participant data (as video) are not practically deidentifiable and are thus not available. Individual team data that underlie the results reported in this article will be made available upon

For peer review only

reasonable request by interested researchers who provide a methodologically sound proposal broadly in-line with the research aims presented in this paper, beginning 3 months and ending 5 years following article publication. Proposals should be directed to c.feather@imperial.ac.uk. To gain access, data requestors will need to sign a data access agreement.

Funding

NA received funding from the Resuscitation Council (UK) which partially funded elements of the simulations and data extraction, with the majority of the funding received from the National Institute for Health Research (NIHR) Imperial Patient Safety Translational Research Centre. Infrastructure support was provided by the NIHR Imperial Biomedical Research Centre (BRC). BDF is also supported by the NIHR Health Protection Research Unit in Healthcare Associated Infections and Antimicrobial Resistance at Imperial College London, in partnership with Public Health England (PHE). The views expressed are those of the authors and not necessarily those of the NHS, PHE, the NIHR or the Department of Health and Social Care.

Figure legends

Figure 1: Overview of study objectives and analyses. *SHERPA = systematic human error reduction and prediction approach

Figure 2: Significance-weighted heat-map of error by process step and SHERPA error mode

Figure 3: The hierarchical task analysis

For peer review only

References

1. World Health Organization. Medication without harm. *B World Health Organ.* 2017;95(5):317-317.
2. Wong IC, Ghaleb MA, Franklin BD, Barber N. Incidence and nature of dosing errors in paediatric medications: a systematic review. *Drug Saf.* 2004;27(9):661-670.
3. Allan EL, Barker KN. Fundamentals of medication error research. *Am J Hosp Pharm.* 1990;47(3):555-571.
4. Kozer E, Seto W, Verjee Z, et al. Prospective observational study on the incidence of medication errors during simulated resuscitation in a paediatric emergency department. *Bmj.* 2004;329(7478):1321.
5. Moreira ME, Hernandez C, Stevens AD, et al. Color-Coded Prefilled Medication Syringes Decrease Time to Delivery and Dosing Error in Simulated Emergency Department Pediatric Resuscitations. *Ann Emerg Med.* 2015;66(2):97-106.e103.
6. Siebert JN, Ehrler F, Combescure C, et al. A Mobile Device App to Reduce Time to Drug Delivery and Medication Errors During Simulated Pediatric Cardiopulmonary Resuscitation: A Randomized Controlled Trial. *J Med Internet Res.* 2017;19(2).
7. Stucki C, Sautter AM, Wolff A, Fleury-Souverain S, Bonnabry P. Accuracy of preparation of i.v. medication syringes for anesthesiology. *Am J Health-Syst Pharm.* 2013;70(2):137-142.
8. Reason J. *Human Error.* Cambridge: University of Cambridge; 1990.
9. Embrey D. Application of SHERPA to Predict and Prevent Use Error in Medical Devices. Paper presented at: International Symposium on Human Factors and Ergonomics in Healthcare: Advancing the cause2014.
10. Lyons M AS, Woloshynowych M, Vincent C. Human reliability analysis in healthcare: A review of techniques. *International Journal of Risk & Safety in Medicine.* 2004;16:223-237.
11. Kirwan B AL. *A guide to task analysis.* London: Taylor and Francis; 1992.
12. National Offshore Petroleum Safety and Environmental Management Authority. Human Reliability Analysis. 2018; https://www.nopsema.gov.au/resources/human-factors/human-reliability-analysis/.
13. Lane R, Stanton NA, Harrison D. Applying hierarchical task analysis to medication administration errors. *Applied ergonomics.* 2006;37(5):669-679.
14. Phipps D, Meakin GH, Beatty PC, Nsoedo C, Parker D. Human factors in anaesthetic practice: insights from a task analysis. *Br J Anaesth.* 2008;100(3):333-343.
15. Joice P, Hanna GB, Cuschieri A. Errors enacted during endoscopic surgery--a human reliability analysis. *Appl Ergon.* 1998;29(6):409-414.
16. BMJ Group and Royal Pharmaceutical Socety. *British National Formulary for Children.* London: Pharmaceutical Press; 2018-2019.
17. Lyons I, Furniss D, Blandford A, et al. Errors and discrepancies in the administration of intravenous infusions: a mixed methods multihospital observational study. *BMJ Qual Saf.* 2018.
18. Shah AN, Frush K, Luo X, Wears RL. Effect of an intervention standardization system on pediatric dosing and equipment size determination: a crossover trial involving simulated resuscitation events. *Arch Pediatr Adolesc Med.* 2003;157(3):229-236.

For peer review only - http://bmjopen.bmj.com/site/about/guidelines.xhtml

19. Garfield S, Reynolds M, Dermont L, Franklin BD. Measuring the severity of prescribing errors: a systematic review. *Drug Saf.* 2013;36(12):1151-1157.
20. Dean BS, Barber ND. A validated, reliable method of scoring the severity of medication errors. *Am J Health Syst Pharm.* 1999;56(1):57-62.
21. Embrey D. SHERPA: A systematic human error reduction and prediction approach. Paper presented at: International Topical Meeting Advances in Human Factors in Nuclear Power Systems1986; American Nuclear Society, La Grange Park, Knoxville Tennessee, IL.
22. McLeod MC, Barber N, Franklin BD. Methodological variations and their effects on reported medication administration error rates. *BMJ Qual Saf.* 2013;22(4):278-289.
23. Lisby M, Nielsen LP, Brock B, Mainz J. How are medication errors defined? A systematic literature review of definitions and characteristics. *Int J Qual Health Care.* 2010;22(6):507-518.
24. Kozer E, Scolnik D, Macpherson A, et al. Variables associated with medication errors in pediatric emergency medicine. *Pediatrics.* 2002;110(4):737-742.
25. Jain S, Basu S, Parmar VR. Medication errors in neonates admitted in intensive care unit and emergency department. *Indian J Med Sci.* 2009;63(4):145-151.
26. Murugan S, Parris P, Wells M. Drug preparation and administration errors during simulated paediatric resuscitations. *Arch Dis Child.* 2018.
27. Porter E, Barcega B, Kim TY. Analysis of medication errors in simulated pediatric resuscitation by residents. *West J Emerg Med.* 2014;15(4):486-490.
28. Children's Acute Transfer Service. Electronic Drug Chart for Referring Hospitals. http://site.cats.nhs.uk/wp-content/uploads/2014/11/dgh_drugcalculator_v1.2.pdf.
29. Patanwala AE, Sanders AB, Thomas MC, et al. A prospective, multicenter study of pharmacist activities resulting in medication error interception in the emergency department. *Ann Emerg Med.* 2012;59(5):369-373.
30. Rothschild JM, Churchill W, Erickson A, et al. Medication errors recovered by emergency department pharmacists. *Ann Emerg Med.* 2010;55(6):513-521.
31. Aldridge VE, Park HK, Bounthavong M, Morreale AP. Implementing a comprehensive, 24-hour emergency department pharmacy program. *Am J Health Syst Pharm.* 2009;66(21):1943-1947.
32. Potts MJ, Phelan KW. Deficiencies in calculation and applied mathematics skills in pediatrics among primary care interns. *Arch Pediatr Adolesc Med.* 1996;150(7):748-752.
33. Fineberg SL, Arendts G. Comparison of two methods of pediatric resuscitation and critical care management. *Ann Emerg Med.* 2008;52(1):35-40 e13.
34. Campagne DD, Young M, Wheeler J, Stroh G. Pediatric Tape: Accuracy and Medication Delivery in the National Park Service. *West J Emerg Med.* 2015;16(5):665-670.
35. Damhoff HN, Kuhn RJ, Baker-Justice SN. Medication preparation in pediatric emergencies: comparison of a web-based, standard-dose, bar code-enabled system and a traditional approach. *J.* 2014;19(3):174-181.

For peer review only - http://bmjopen.bmj.com/site/about/guidelines.xhtml

For peer review only

For peer review only - http://bmjopen.bmj.com/site/about/guidelines.xhtml

Figure 1. Overview of study objectives and analyses

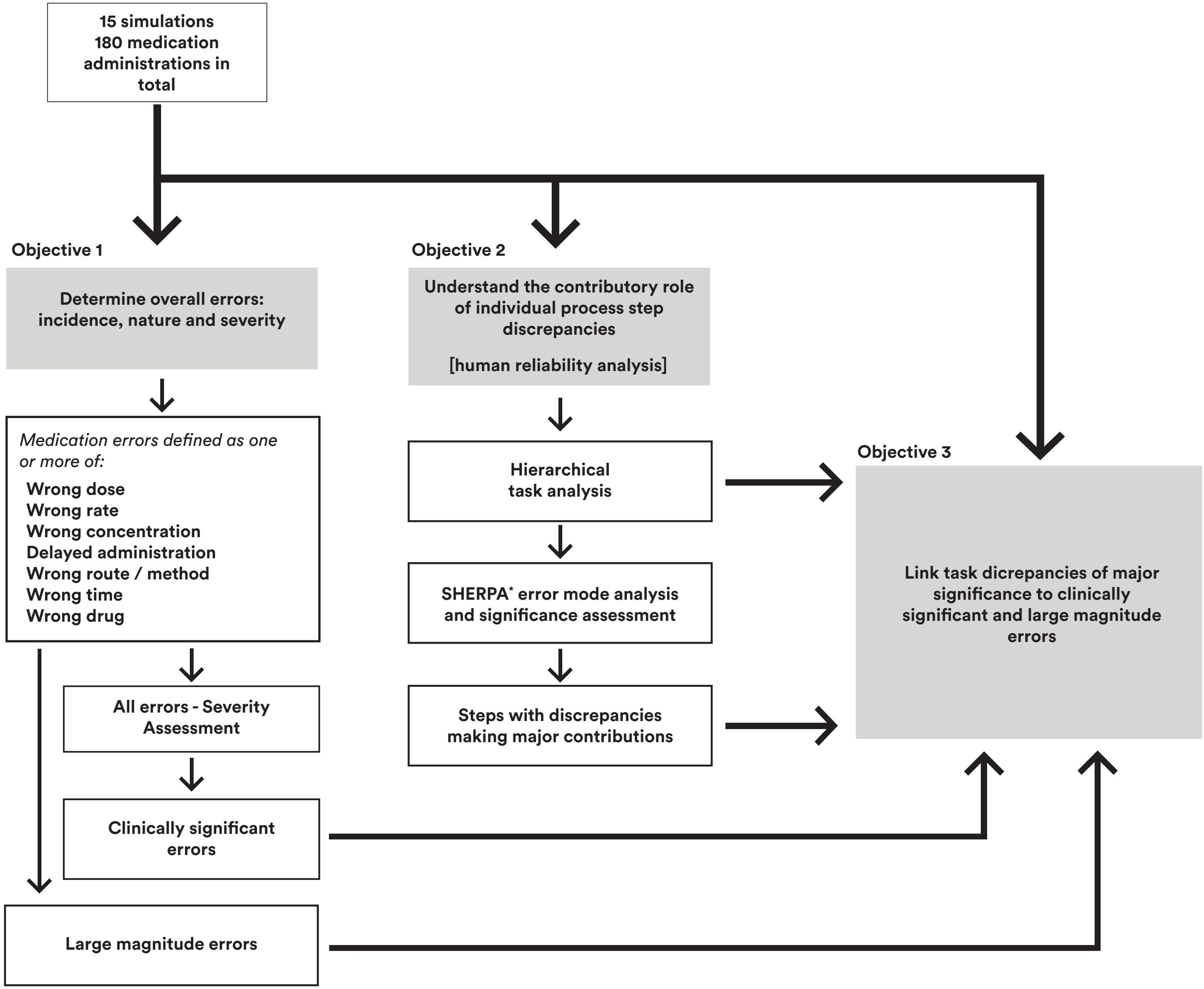

*SHERPA = systematic human error reduction and prediction approach

Figure 2. Significance-weighted heat-map of error by process step and SHERPA error mode

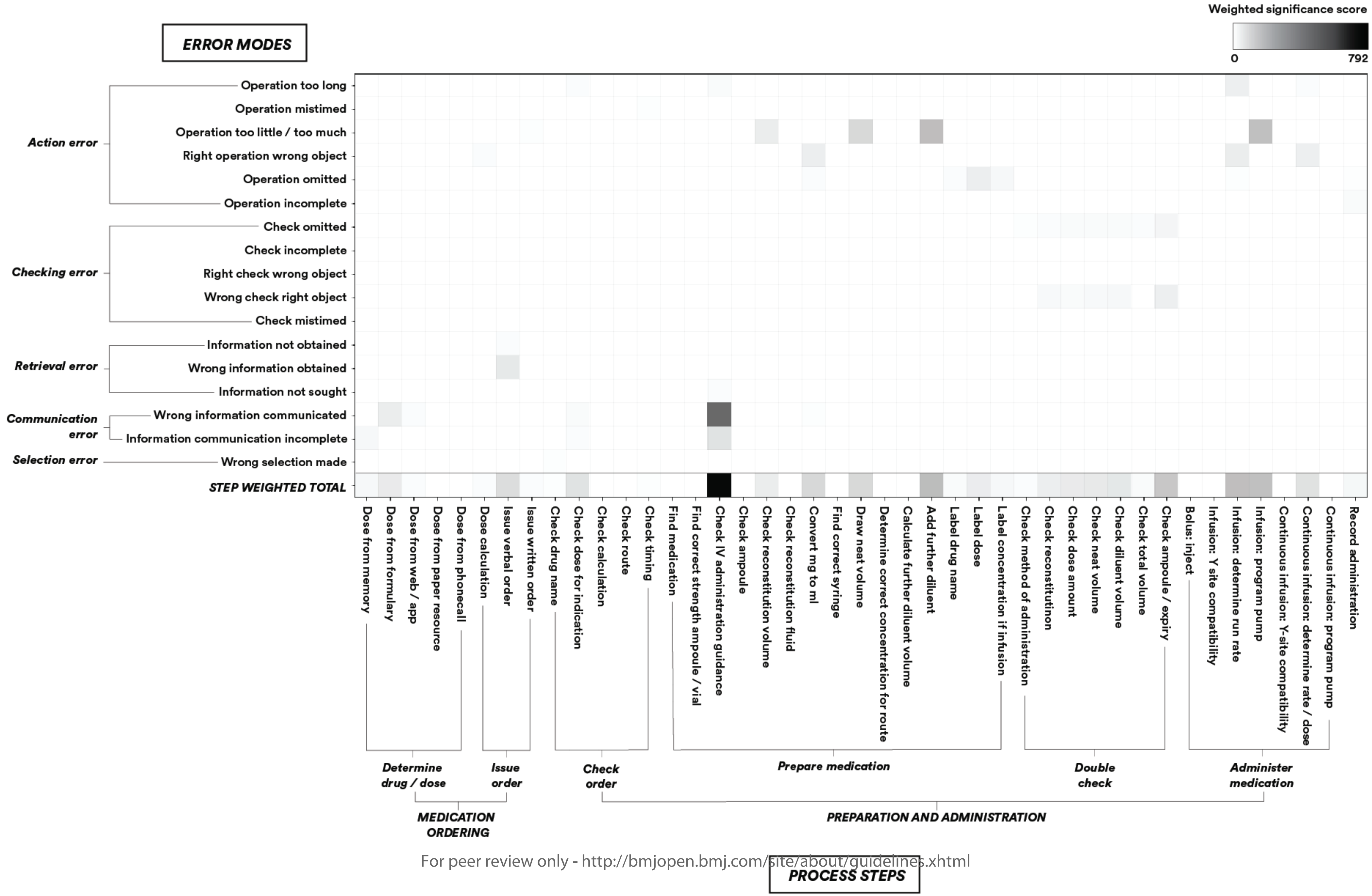

For peer review only - http://bmjopen.bmj.com/site/about/guidelines.xhtml

Figure 3. The hierarchical task analysis

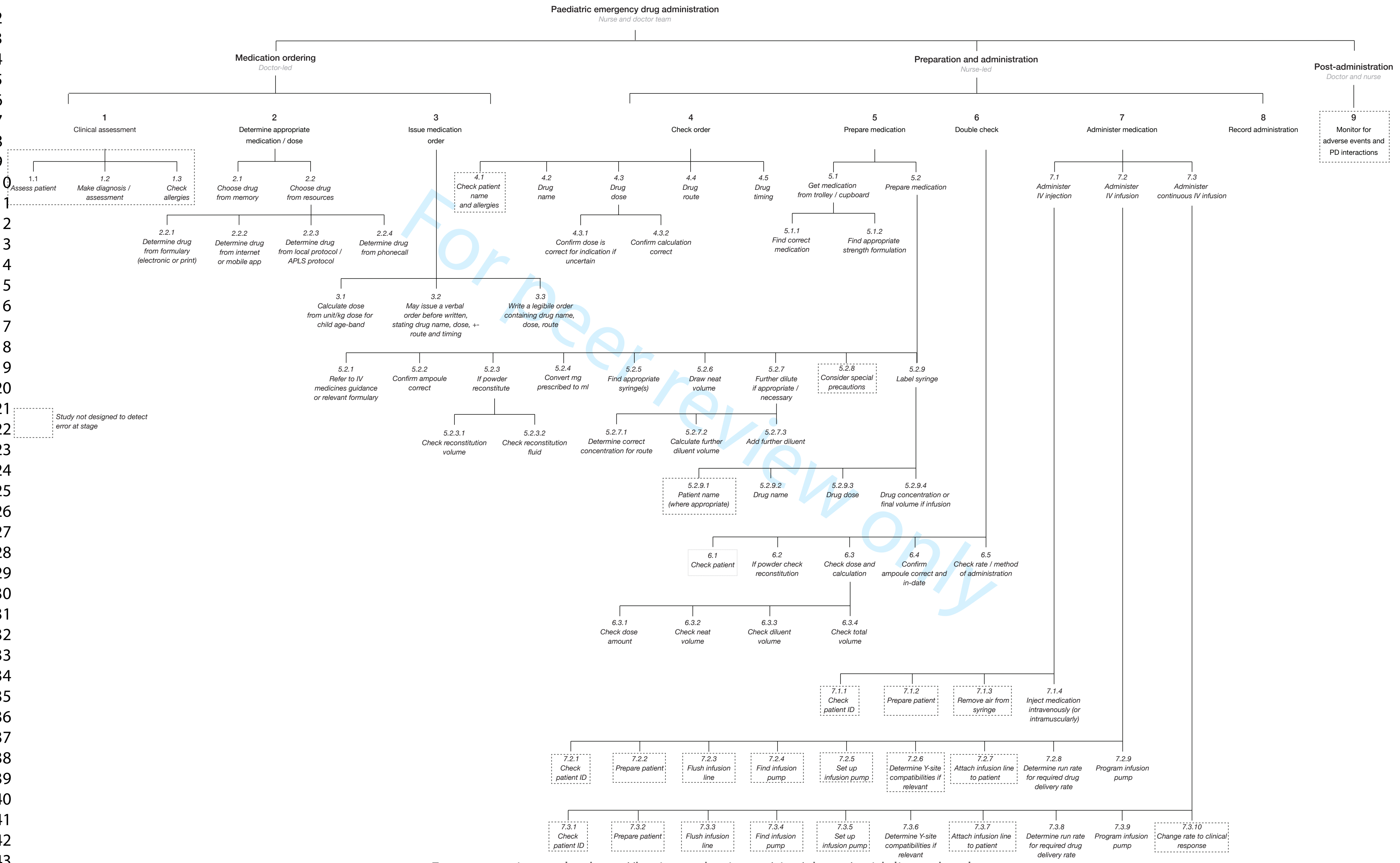

For peer review only

Appendices

Appendix 1. Case vignettes, expected medications and acceptable dose ranges

Case 1: Fitting Child

An 8-month-old, 8kg, previously well infant presents in prolonged status epilepticus with a two day history of coryza and one day history of lethargy and fever. On assessment the infant is having a generalised tonic clonic seizure and is febrile.

Teams were expected to treat the child for status epilepticus as per the standard paediatric guidelines, as well as add appropriate antimicrobial cover for presumed meningo-encephalitis. Additionally, all electrolyte abnormalities, including hypoglycaemia (glucose level initially 2.2), should be corrected as they would contribute to seizure activity.

In this case the following drugs were expected to be administered intravenously (in order of clinical priority).

- Lorazepam two doses (0.1mg/kg)
- 10% dextrose for hypoglycaemia (2-5ml/kg) [higher dose accepted as per British National Formulary]
- Phenytoin, loading dose (20mg/kg)
- Rapid Sequence Induction
    - Thiopentone (2-4mg/kg)
    - Suxamethonium (2mg/kg)
- Ceftriaxone (80-100mg/kg)
- Aciclovir (500mg/m^2 = 26mg/kg in this case)
- Clarithromycin (7.5mg/kg)
- Sedation and paralysis post intubation
    - Morphine infusion (10-40mcg/kg/hour)
    - Midazolam infusion (1-6mcg/kg/min)
    - Vecuronium infusion (0.8-3mcg/kg/min)

For peer review only

Case: Sepsis with shock

A 10-month-old, 9kg, previously well and fully immunised infant presents with shock and presumed meningococcal septicaemia. The infant has a two-day history of cough, coryza and fever, and has developed a spreading rash all over his body. On assessment the infant is floppy, only responsive to pain, with a spreading non-blanching purpuric rash all over his torso. He is febrile (40°C) with cold extremities and extremely poor perfusion. He has a sinus tachycardia with an unrecordable non-invasive blood pressure.

Teams were expected to initiate aggressive management of shock with fluids and vasoactive drugs in order to stabilise the infant. Early administration of appropriate antimicrobials was expected, as was the correction of metabolic derangements are also required.

In this case this would mean the following drugs should be administered (in order of clinical priority). Intraosseous access was expected to be established prior to successful intravenous cannulation.

- Ceftriaxone (80-100mg/kg)
- 10% dextrose for hypoglycaemia (2-5ml/kg) [higher dose accepted as per British National Formulary]
- Fluid resuscitation (fluid boluses were not measured for this study)
- Adrenaline infusion (0.1-0.5mcg/kg/min)
    - or
- Dopamine infusion (5-20mcg/kg/min) + adrenaline infusion
- Calcium for hypocalcaemia (0.11mmol/kg)
- Sodium Bicarbonate for acute mixed metabolic/respiratory acidosis (1ml/kg of 8.4%)
- Rapid Sequence Intubation
    - Ketamine (1-2mg/kg)
    - Suxamethonium (2mg/kg)
- Sedation and paralysis post intubation
    - Morphine infusion (10-40mcg/kg/hour)

For peer review only

- Midazolam infusion (1-6mcg/kg/min)
- Vecuronium infusion (0.8-6mcg/kg/min)

For peer review only

Appendix 2. Detailed operational definitions and explanation

Main outcome measures and definitions of error

Medication errors

- **Administrations were classed as <u>dosing errors</u> where:**
  - For drugs given by **injection** (bolus), there was a 10% or greater deviation from the recommended dosing range (DRDR). The DRDR was calculated as the absolute value of the percentage deviation from the recommended dose or dose range.
  - For medications administered as **infusions**, there was a 10% or greater deviation in either the DRDR or the deviation from the recommended dosing rate (DRDRate). The DRDRate was calculated as the absolute value of the percentage deviation from the recommended rate of administration for each medication.
  - For medications administered as **continuous infusions**, there was a 10% or greater deviation from the DRDRate.

- **<u>Timing errors</u>**
  - There is no study to describing acceptable times to medication administration for individual drugs in paediatric emergencies that it is possible to use as a performance benchmark. This lack of clarity is further complicated by the fact that after the successful preparation of a medication to be administered, it is regularly the case that practical issues such as line availability introduce delays. To make an assessment of prolonged administrations, therefore, times to drug 'ready for delivery' (tDRD) were used as a point of reference. The tDRD was calculated as the sum of the elapsed times during which the doctors were seeking medication information in resources with the total nurse-led preparation time. The timings used in this study are shown in more detail in the accompanying figure below.

For peer review only

- tDRD's were considered prolonged where a particular team took greater than 200% of the median tDRD for that specific drug across the entire study sample.
- Drugs given at the wrong time were classed as *'Wrong time'* errors
- Overall delays in tDRD due to clinical reasons as determined by observation, for example interruptions in order to perform critical tasks or give more important medications, were excluded as errors.

Figure 1. Timing definitions used for overall medication errors and individual process steps

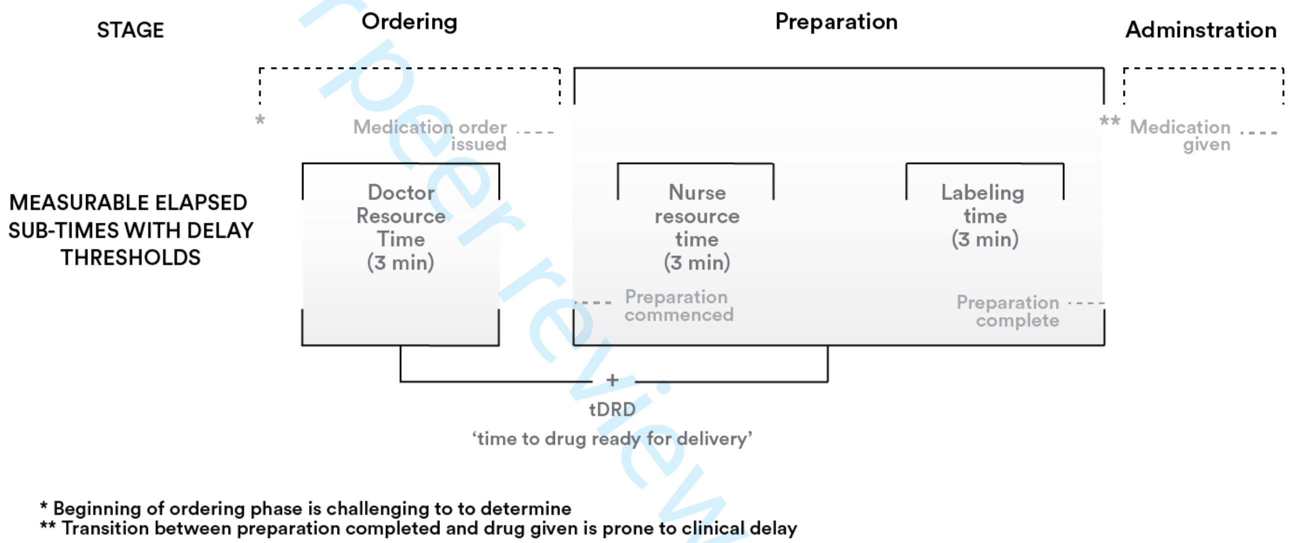

- **Other errors**
  - In addition, medication administrations were considered erroneous overall where
  - There was deviation from the recommended concentration (DRC) of greater than 10% from the suggested concentration in the IV medicines guidance.
  - There was a wrong drug error, a deviation from the suggested route or method of administration.

- All administrations **considered erroneous overall** were presented for severity assessment.

At the level of the task: the HRA / SHERPA framework

1. Task analysis

A hierarchical task analysis (HTA) of the procedure for the administration of bolus as well as medications administered by infusion was undertaken, adapting the Lane framework [3] for local emergency practice. The task analysis divided the procedure into three 'doctor-led' and five 'nurse-led' parent tasks, and each of these was broken down hierarchically into exhaustive sub-tasks. The HTA was assessed for face validity by five senior nurses in the study hospital.

2. Error mode categorisation and assessment

To understand the steps that were prone to introducing error, we used a generic human error taxonomy based on the SHERPA External Error Modes [4], with the addition of one additional error mode. Each step discrepancy was coded against this framework.

- **Coding thresholds for specific steps**
  - Step discrepancies that **made a contribution of more than 5% DRDR or DRDRate to an overall administration were coded as erroneous**. The lower threshold was chosen to identify causes for errors that were the result of cumulative step discrepancies.
  - There is no guidance with respect to acceptable times for retrieval of medication information from print or electronic resources. Guided by the expert panel, it was decided to consider times for either doctors or the nursing team to retrieve the appropriate information prolonged where they took more than 3 mins at any individual resource.
  - The following were considered the minimum requirements for safe labelling practice:
    - For IV injections: drug name and dose

For peer review only

- For IV infusions and continuous infusions: drug name, dose and strength of prepared mixture after dilution.

Where there were more than two discrepancies at a single step for a specific administration, the nurse evaluator made a subjective assessment of which error had the greater overall consequence and assigned an error mode to that discrepancy only.

3. Downstream or dependent errors

Root cause analysis (RCA) is a technique used to attempt to establish how and why an incident occurred [5]. While this study was not a typical RCA, to capture only 'root-cause' system vulnerabilities, steps where an action was performed correctly, but which perpetuated a previous error were not classed as erroneous. For example, a drug may be prescribed incorrectly, but then drawn up correctly with respect to the prescription. In this case, a prescription error would be recorded, while the drawing up step would not be classed as erroneous. Other errors which are independent of the prescription error, for example failure to double check the medication, were coded as discrepancies if present.

4. Significance assessment for individual step discrepancies

Medication preparation and administration is regularly subject to many minor sub-step discrepancies which may not individually, or even in combination, result in clinically significant error reaching the patient. In order to understand those sub-task errors which contributed to errors overall, significance scores were attributed to each coded sub-step error as follows:

- **No contribution**: discrepancy made no contribution to overall error
- **Minor contribution**: some contribution to overall error. A 5% threshold (for DRDR, DRDRate, DRC) was used to detect task discrepancies contributing in combination to significant errors.
- **Major contribution**: a principally contributory sub-task discrepancy that led directly to the medication error

For peer review only

References

1. Garfield, S., et al., *Measuring the severity of prescribing errors: a systematic review.* Drug Saf, 2013. **36**(12): p. 1151-7.
2. Dean, B.S. and N.D. Barber, *A validated, reliable method of scoring the severity of medication errors.* Am J Health Syst Pharm, 1999. **56**(1): p. 57-62.
3. Lane, R., N.A. Stanton, and D. Harrison, *Applying hierarchical task analysis to medication administration errors.* Applied ergonomics, 2006. **37**(5): p. 669-79.
4. Embrey, D. *SHERPA: A systematic human error reduction and prediction approach*. in *International Topical Meeting Advances in Human Factors in Nuclear Power Systems*. 1986. American Nuclear Society, La Grange Park, Knoxville Tennessee, IL.
5. United States Department of Veteran Affairs., *Root cause analysis*. VA National Center for Patient Safey, 2015. http://www.patientsafety.va.gov/professionals/onthejob/rca.asp

For peer review only

STROBE Statement

	Item No	Recommendation
**Title and abstract**	1	(*a*) Indicate the study's design with a commonly used term in the title or the abstract **[Title, abstract and methods section of manuscript]**
		(*b*) Provide in the abstract an informative and balanced summary of what was done and what was found **[See results section of abstract]**
**Introduction**		
Background/rationale	2	Explain the scientific background and rationale for the investigation being reported **[page 2]**
Objectives	3	State specific objectives, including any prespecified hypotheses **[page 2]**
**Methods**		
Study design	4	Present key elements of study design early in the paper **[pages 3 and 4]**
Setting	5	Describe the setting, locations, and relevant dates, including periods of recruitment, exposure, follow-up, and data collection **[page 3]**
Participants	6	(*a*) Give the eligibility criteria, and the sources and methods of selection of participants. Describe methods of follow-up **[page 3]**
		(*b*) For matched studies, give matching criteria and number of exposed and unexposed **[N/A]**
Variables	7	Clearly define all outcomes, exposures, predictors, potential confounders, and effect modifiers. Give diagnostic criteria, if applicable **[pages 4 and 5]**
Data sources/ measurement	8*	For each variable of interest, give sources of data and details of methods of assessment (measurement). Describe comparability of assessment methods if there is more than one group **[page 4]**
Bias	9	Describe any efforts to address potential sources of bias **[page 4]**
Study size	10	Explain how the study size was arrived at **[pages 3 and 8]**
Quantitative variables	11	Explain how quantitative variables were handled in the analyses. If applicable, describe which groupings were chosen and why **[pages 6 and 7]**
Statistical methods	12	(*a*) Describe all statistical methods, including those used to control for confounding **[page 6 and 7]**
		(*b*) Describe any methods used to examine subgroups and interactions **[page 6 and 7]**
		(*c*) Explain how missing data were addressed **[N/A]**
		(*d*) If applicable, explain how loss to follow-up was addressed **[N/A]**
		(*e*) Describe any sensitivity analyses **[N/A]**
**Results**		
Participants	13*	(a) Report numbers of individuals at each stage of study—eg numbers potentially eligible, examined for eligibility, confirmed eligible, included in the study, completing follow-up, and analysed **[page 7 and table 1]**
		(b) Give reasons for non-participation at each stage **[N/A]**
		(c) Consider use of a flow diagram **[N/A – described in text]**
Descriptive data	14*	(a) Give characteristics of study participants (eg demographic, clinical, social) and information on exposures and potential confounders **[page 7 and 8 and table 1]**
		(b) Indicate number of participants with missing data for each variable of interest **[table 1]**
		(c) Summarise follow-up time (eg, average and total amount) **[N/A]**
Outcome data	15*	Report numbers of outcome events or summary measures over time **[N/A]**
Main results	16	(*a*) Give unadjusted estimates and, if applicable, confounder-adjusted estimates and

For peer review only - http://bmjopen.bmj.com/site/about/guidelines.xhtml

		their precision (eg, 95% confidence interval). Make clear which confounders were adjusted for and why they were included **[N/A]**
		(*b*) Report category boundaries when continuous variables were categorized **[pages 3 and 4, tables 2 and 3]**
		(*c*) If relevant, consider translating estimates of relative risk into absolute risk for a meaningful time period **[N/A]**
Other analyses	17	Report other analyses done—eg analyses of subgroups and interactions, and sensitivity analyses **[pages 8, 9, 10]**
**Discussion**		
Key results	18	Summarise key results with reference to study objectives **[page 14]**
Limitations	19	Discuss limitations of the study, taking into account sources of potential bias or imprecision. Discuss both direction and magnitude of any potential bias **[page 15]**
Interpretation	20	Give a cautious overall interpretation of results considering objectives, limitations, multiplicity of analyses, results from similar studies, and other relevant evidence **[page 15 and 16]**
Generalisability	21	Discuss the generalisability (external validity) of the study results **[page 14]**
**Other information**		
Funding	22	Give the source of funding and the role of the funders for the present study and, if applicable, for the original study on which the present article is based **[within acknowledgements on title page]**

*Give information separately for exposed and unexposed groups.

Note: An Explanation and Elaboration article discusses each checklist item and gives methodological background and published examples of transparent reporting. The STROBE checklist is best used in conjunction with this article (freely available on the Web sites of PLoS Medicine at http://www.plosmedicine.org/, Annals of Internal Medicine at http://www.annals.org/, and Epidemiology at http://www.epidem.com/). Information on the STROBE Initiative is available at http://www.strobe-statement.org.

For peer review only
